# Pterostilbene promotes mitochondrial apoptosis and inhibits proliferation in glioma cells

**DOI:** 10.1038/s41598-021-85908-w

**Published:** 2021-03-18

**Authors:** Haijun Gao, Ziqiang Liu, Weidong Xu, Qunhui Wang, Chaochao Zhang, Yaonan Ding, Weiguang Nie, Jiacheng Lai, Yong Chen, Haiyan Huang

**Affiliations:** 1 Department of Neurosurgery, First Hospital of Jilin University, Changchun, 130021 Jilin Province China; 2 Department of Neurosurgery, Fenyang Hospital of Shanxi Province, Fengyang, 032200 Shanxi Province China

**Keywords:** CNS cancer, Cancer

## Abstract

Glioma is the most general primary and lethal intracranial malignant tumor. Pterostilbene (PTE), an analog of stilbene and resveratrol, has attracted attention in recent years due to its significant antitumor activity in multiple solid tumors; however, its effect on drug-resistant glioma cells and the underlying mechanism have not yet been reported. In this study, we found that pterostilbene inhibited proliferation, induced intrinsic mitochondria-mediated apoptosis and caused S phase arrest, inhibited migration and excessive invasion in glioma cells. Pretreatment with the pan-caspase-inhibitor Z-VAD-FMK attenuated the PTE-induced apoptosis of glioma cells. Moreover, PTE significantly increased the production of reactive oxygen species (ROS) and reduce the mitochondrial membrane potential (MMP). Inhibition of ROS with *N*-acetyl-l-cysteine not only rescued PTE-induced reduction of cellular viability but also prevented glioma cell apoptosis. We also discovered ERK 1/2 and JNK signaling pathways were activated by PTE and contributed to induce glioma cell apoptosis. In addition, specific inhibitors of ERK 1/2 and JNK attenuated PTE-induced apoptosis. Besides, PTE significantly reduced tumor volume and prolonged median survival of tumor-bearing rats in vivo. In summary, the results of this study indicate that the anti-tumor effect of PTE on glioma cells may provide a new treatment option for glioma patients.

## Introduction

Glioma originates from glial and neuronal cells of the nervous system and is the most common primary intracranial tumor^1^. Glioblastoma multiforme (GBM) is the most aggressive primary malignancy in the adult central nervous system, accounting for approximately 54% of all gliomas^2^. Current glioblastoma treatment mainly involves surgery supplemented by comprehensive postoperative radiotherapy and chemotherapy treatments^3^. Although recent progress and improvements in comprehensive treatment methods are emerging, overall outcomes for glioblastoma remain unsatisfactory. Survival time for most patients has not changed significantly—especially for GBM patients, in which the five-year survival rate does not exceed 5%^4^. Consequently, more effective treatment methods are urgently needed to improve outcomes in glioblastoma.

**Figure 1:**
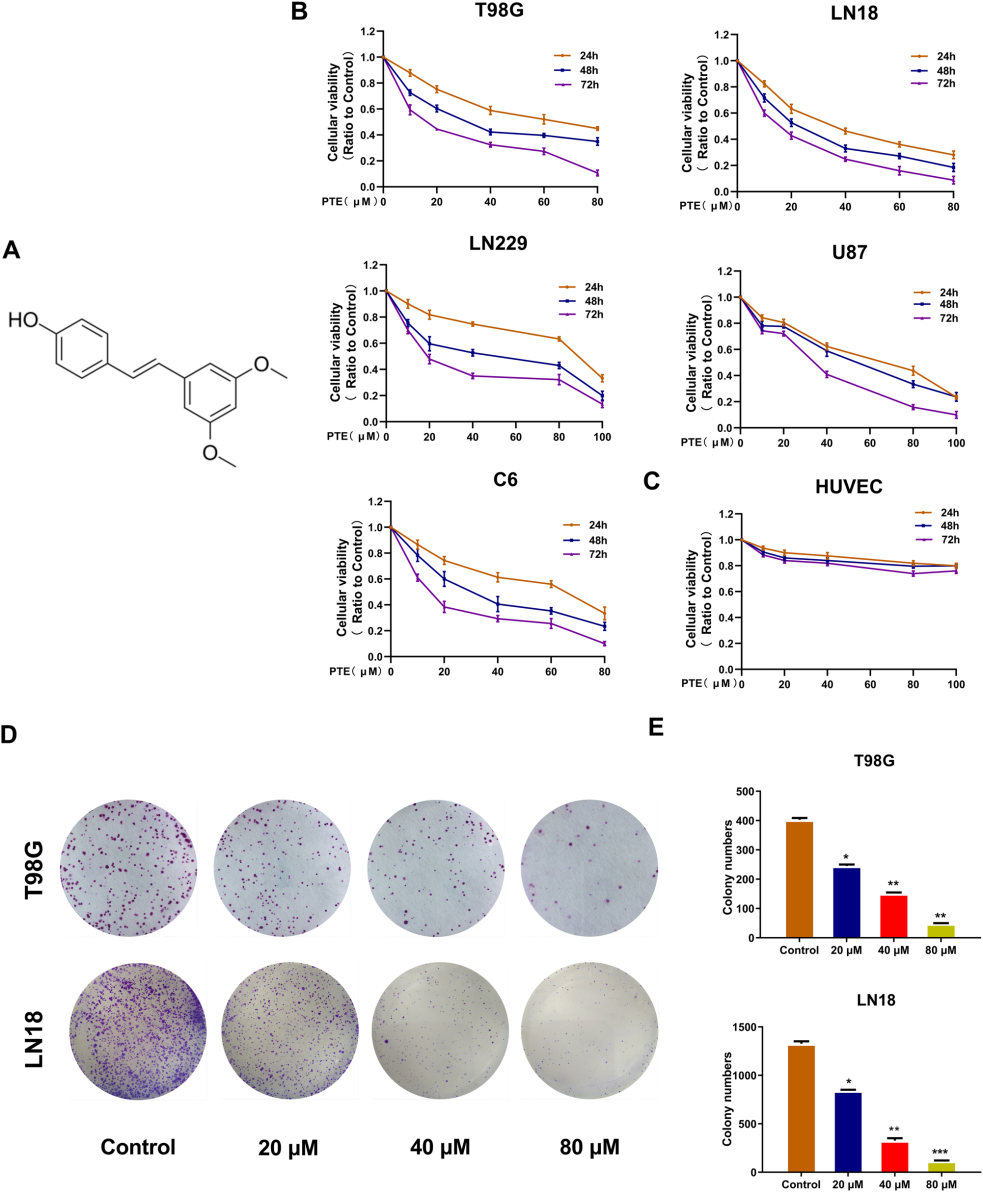
Pterostilbene (PTE) inhibited the viabilities of glioma cell lines. (**A**)The chemical structure of PTE. (**B**) and (**C**) T98G, LN18, U87, LN229 glioma cells and HUVEC cell were treated with PTE for 24, 48 and 72 h. Cell viability was measured by MTT assay. (**D**) and (**E**) The long-term effect of PTE on the proliferative capacity of glioma cells was carried out by colony formation assays. Representative images of colony formation and analyses of the colony numbers observed in T98G and LN18 cells after 10 days of treatment with PTE at the indicated concentrations. Data are presented as the mean ± standard deviation (SD); n = 3; *p < 0.05, **p < 0.01 and ***p < 0.001, compared to the vehicle control group. (GraphPad Prism 8.0 https://www.graphpad.com/).

With the development of comprehensive treatment for glioblastoma in recent years, the anti-cancer effects of natural products and phytochemicals commonly used in traditional Chinese medicine continue to attract widespread attention^5^. Some medicines show obvious anti-tumor properties and low toxicity, offering potential options for glioblastoma treatment in the future^6^. Pterostilbene (trans-3, 5-dimethoxy-4-hydroxystilbene, PTE) (Fig. 1A), is a dimethyl analog of resveratrol and is mainly found in blueberries and grapes^7^. Due to the lipophilicity of its two methoxyl groups, PTE has better bioavailability and longer half-life after ingestion, suggesting that PTE has greater clinical application potential^8^. Indeed, PTE has shown a wide range of biological functions, including antitumor, antioxidant, anti-inflammatory, apoptotic, cardiovascular protective, anti-proliferative, and antibacterial^9^. In recent years, several studies report that PTE renders its anticancer effect by inhibiting proliferation and inducing apoptosis in various types of cancers, including breast^10,11^, pancreatic^12,13^, prostate^14,15^, bladder^16^, gastric carcinoma^17^, colorectal^18,19^, lung^20,21^, and oral cancer^22^, as well as hepatocellular carcinoma^23–25^ and leukemia^26,27^. Moreover, in 2017, Zielinska-Przyjemska and his colleagues evaluated the effect of resveratrol and its analogs (Pterostilbene and 3,5,4′-trimethoxystilbene) along with tannic acid and discovered that PTE acted as a more efficient inhibitor of glioma cell proliferation and apoptosis^28^.

Various cellular metabolic processes are related to reactive oxygen species (ROS), such as transcription factor activation, gene expression, cell differentiation and proliferation29. However, excessive ROS can also cause cell damage, such as the oxidation of cardiolipin in the center of the mitochondrial membrane and the reduction of mitochondrial membrane potential (MMP), which promotes apoptosis^30,31^. In recent years, studies have shown that ROS can activate mitogen-activated protein kinases (MAPK) families. MAPK families regulate lots of cellular processes, including cell growth, proliferation, differentiation, survival and death^32^. At least three conventional MAPKs are expressed in mammals, including extracellular signal-regulated kinase (ERK), c-JUN N-terminal kinase (JNK) and p38, and the dysregulation of MAPK is closely related to the occurrence of many human tumors^33^. Although the activation of JNK and p38 is related to cell apoptosis under environmental stress conditions, especially under oxidative damage, it is generally believed that the activation of ERK induced by mitogens, growth factors and cytokines will trigger survival signals^34^. However, recent studies have shown that ERK activation can also lead to tumor cell apoptotic death, which is a response to various anticancer drugs^35^. For example, Wakimoto^36^ et al. a found that pterostilbene can inhibit the proliferation of triple-negative breast cancer cells and promote their apoptosis, which may cause G_0_/G_1_ cell cycle arrest through strong and sustained activation of the ERK pathway.

Herein, we determined the potential anti-tumor effects of PTE in glioma cells both in vitro and in vivo. Furthermore, we explored the molecular mechanisms underlying glioma cell apoptosis upon treatment with PTE, including the relationship between conventional MAPKs and ROS, the elucidation of which may provide novel applications for PTE as a potential drug for glioma in the future.

## Results

### Pterostilbene inhibited viability of glioma cell lines

To explore the effects of pterostilbene on malignant glioma cells, T98G, LN18, LN229, U87, and C6 glioma cells were treated with various concentrations of PTE for 24, 48 and 72 h. The MTT assay results (Fig. 1B) showed that PTE inhibited the growth of glioma cells in a dose- and time- dependent manner. Thus, the 50% growth-inhibitory concentration (IC50) values of PTE treatment for 48 h were 32.93 μM on T98G cells, 22.30 μM on LN18 cells, 46.18 μM on U87 cells, 37.56 μM on LN229 cells, and 30.10 μM on C6 cells. Meanwhile, to investigate the toxicity of PTE on normal cells, HUVECs were selected and treated with the same concentration and time. MTT assay results showed, the inhibitory effect of PTE on HUVECs was significantly lower than the selected glioma cells described above (Fig. 1C). As a consequence, we chose the sensitive cell lines T98G and LN18 as candidate cell lines for the next study and 48 h as the duration of PTE treatment.

Moreover, to evaluate the long-term effect of PTE on the proliferative capacity of glioma cells, we carried out colony formation assays. The results showed that colony formation in the T98G and LN18 cells was significantly inhibited after incubation with a low-dose (20 μM) for 10 days and the inhibition acted in a dose-dependent manner (Fig. 1D,E).

### Pterostilbene treatment promoted caspase activation and induced apoptosis in glioma cells

**Figure 2:**
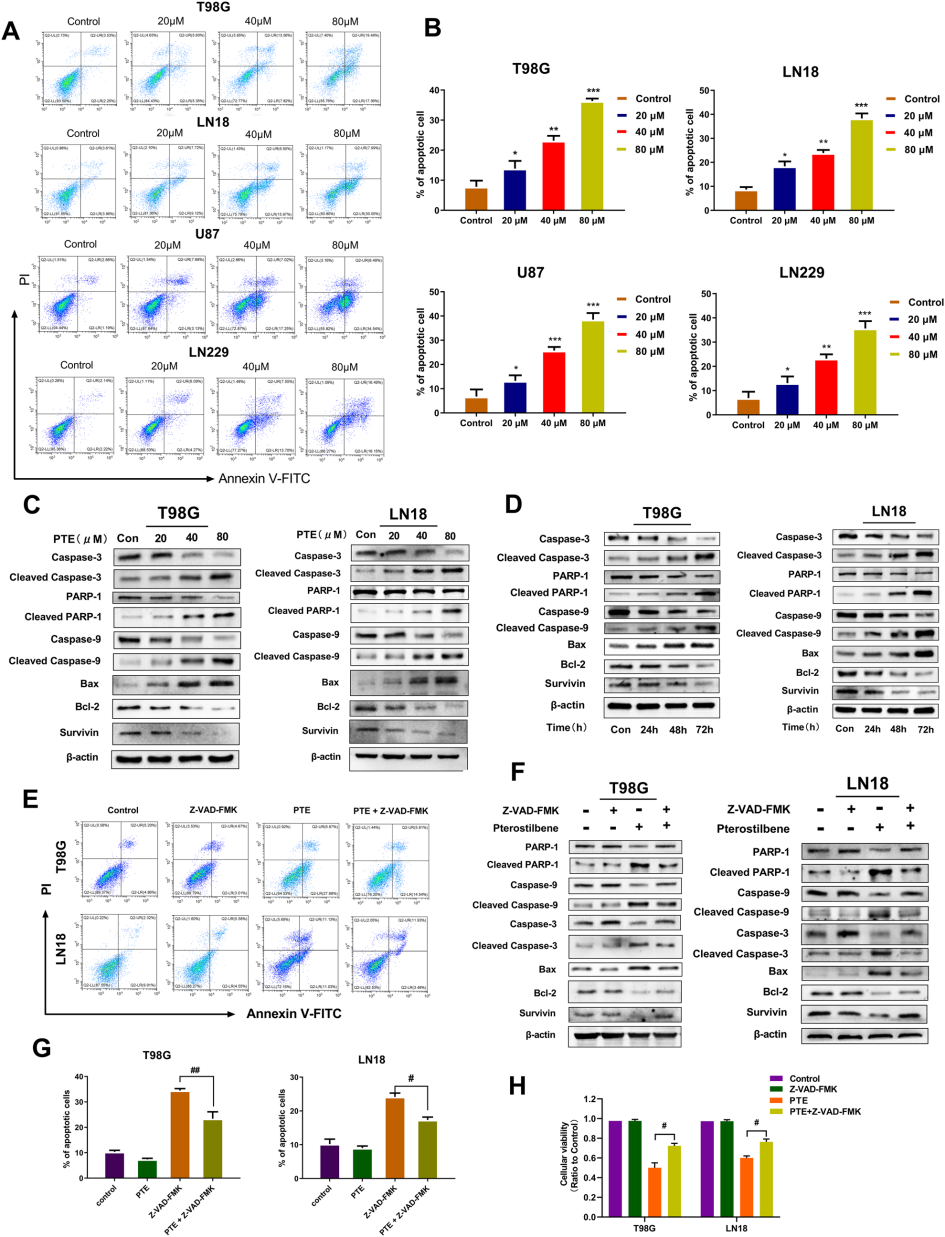
Pterostilbene induced apoptosis and promoted caspase activation in glioma cells. (**A**) T98G, LN18, U87 and LN229 glioma cells were treated with PTE for 48 h, early and late apoptotic cells were analyzed using flow cytometry combined with Annexin V/PI double staining. (**B**) Histogram showed the mean percentage of apoptosis. (**C**) The expression levels of caspase-3,cleaved caspase-3, PARP-1, cleaved PARP-1, caspase-9, cleaved caspase-9, Bax, Bcl-2 and Survivin were detected by western blotting after treated with PTE for 48 h in T98G and LN18. (**D**) The apoptosis-related proteins (including Caspase-3 and 9, cleaved Caspase-3 and 9,PARP-1 and cleaved PARP-1, Bax, Bcl-2 and Survivin) were tested after treated with PTE (40 µM) for 24 h, 48 h, 72 h in T98G and LN18 glioma cells. (**E**) T98G and LN18 cells were pre-incubated with Z-VAD-FMK (50 µM) for 2 h and then treated with PTE (40 µM) for 48 h. Apoptotic cells were analyzed by flow cytometry. (**F**) The expression levels of caspase-3,cleaved caspase-3, PARP-1, cleaved PARP-1, caspase-9, cleaved caspase-9, Bax, Bcl-2 and Survivin were detected by western blotting in T98G and LN18 cells pre-incubated with Z-VAD-FMK. (**G**) Histogram showed the mean percentage of apoptosis cells treated with PTE that pre-incubated with or without Z-VAD-FMK. (**H**)Z-VAD-FMK (50 µM) was added to T98G and LN18 cells 2 h before PTE treatment. After 48 h, cell viability was determined by MTT assay. β-actin was used as a loading control. Data are presented as the mean ± standard deviation (SD); n = 3; (*p < 0.05, **p < 0.01 and ***p < 0.001, compared to the vehicle control group); (^#^*p* < 0.05, ^##^*p* < 0.01, PTE + Z-VAD-FMK vs PTE).

To explore whether the inhibition of PTE on the proliferation of glioma cells is related to the induction of apoptosis, T98G, LN18, U87 and LN229 cells were treated with different concentrations of PTE for 48 h and flow cytometry with FITC Annexin V/PI stain was used to detect apoptosis. Our data exhibited that the proportion of early and late apoptotic cells, especially early apoptotic cells, were significantly increased dose-dependently (Fig. 2A,B).

**Figure 3:**
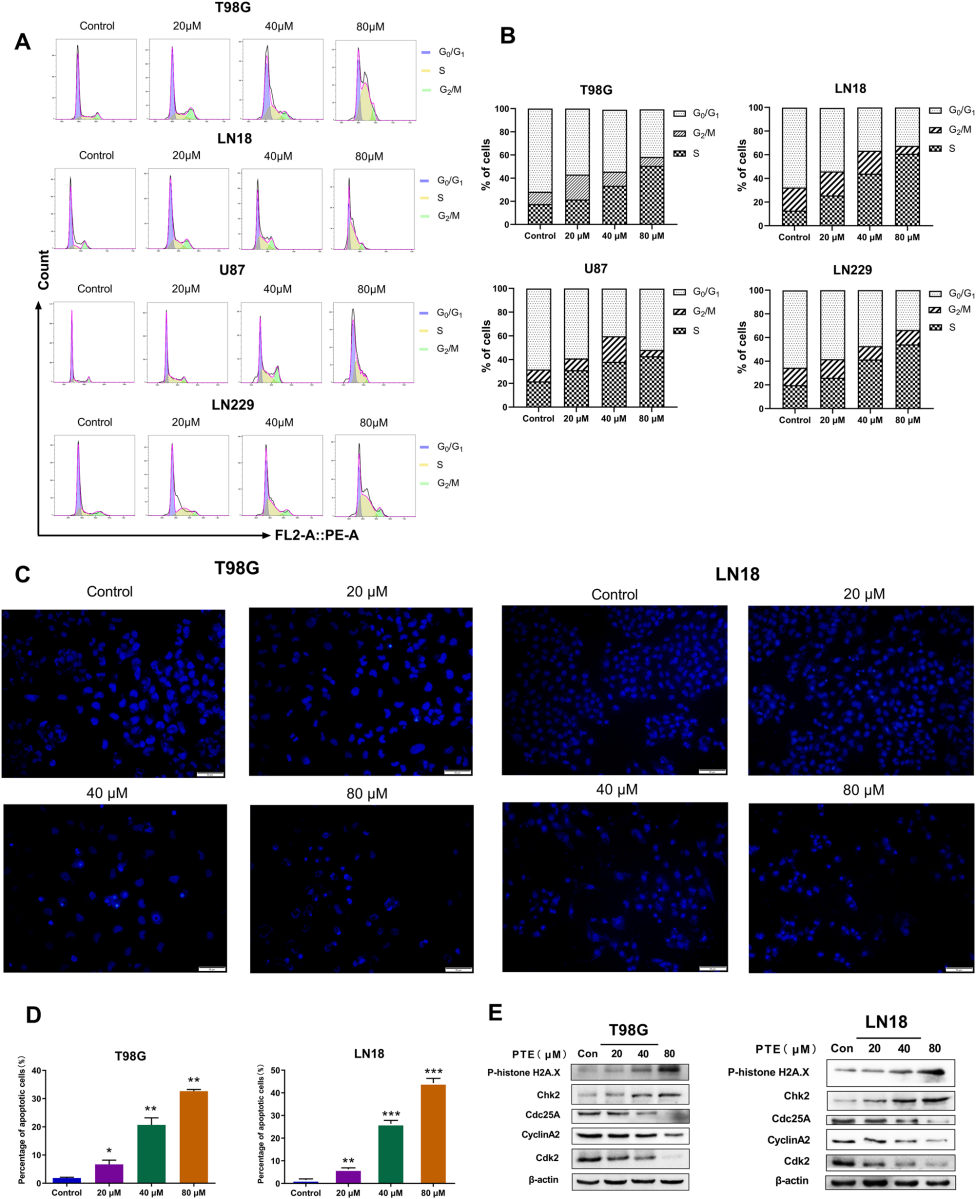
Pterostilbene induces S phase cell cycle arrest in glioma cell lines. (**A**) T98G, LN18, U87 and LN229 glioma cells were treated with PTE (0, 20, 40 and 80 µM) for 48 h followed by propidium iodide staining and cell cycle distribution was analyzed by flow cytometry. (**B**) Histograms showed the percentage of T98G, LN18, U87 and LN229 glioma cells in G_0_-G_1_, S, and G_2_ phases. (**C**)T98G and LN18 cells were treated with PTE (0, 20, 40, 80 µM) for 48 h before staining with 10 mg/L Hoechst 33342. The representative images under a fluorescence microscope (20 ×) showed the Morphological changes. (**D**) Percentages of apoptotic cells in PTE-treated T98G and LN18 cells were analyzed. (**E**) The levels of cell cycle-associated protein expressions (phospho-Histone H2A.X, cdc25A, CDK2, Chk2 and Cyclin A2) were analyzed by western blotting following exposure with PTE for 48 h in T98G and LN18 cells. β-actin was used as a loading control. Data are presented as the mean ± standard deviation (SD); n = 3; *p < 0.05, **p < 0.01 and ***p < 0.001, compared with the vehicle control group.

To further confirm these results, the morphologic changes in T98G and LN18 cells were detected by Hoechst 33342 staining. Hoechst 33342 staining is sensitive to DNA and is used to evaluate the changes in the nucleus morphology. When cells undergo apoptosis, there will be typical apoptotic features, namely nuclear condensation/chromatin fragmentation and apoptotic bodies. After Hoechst 33342 staining, bright blue fluorescent spots will appear under the fluorescence microscope^37^. As shown in Fig. 3C, the control group showed uniform blue staining of nuclei, with the gradual increase of PTE concentration, the glioma cells undergoing apoptosis gradually became smaller, and more enhanced blue bright spots gradually appeared under the fluorescence microscope. Moreover, in Fig. 3D we also found that the proportion of apoptotic cells also increased significantly with the increase of PTE treatment.Next, our team investigated the expression levels of cleaved caspase-3, cleaved caspase-9 and cleaved PARP-1 in LN18 and T98G cells after 48 h PTE treatment. Western blotting (Fig. 2C) indicated that PTE induced a concentration-dependent increase in cleaved PARP-1, cleaved caspase-3 and cleaved caspase-9. The increased cleaved PARP-1 further confirmed the activation of caspase-3. Besides, we also evaluated the pro-apoptotic protein Bax and anti-apoptotic proteins Bcl-2, two mitochondrial-related apoptosis regulators. Our results indicate that Bax was increased significantly, while Bcl-2 and Survivin were decreased significantly in a dose-dependent manner after PTE treatment. (Fig. 2C). Moreover,we also tested the effect of PTE on apoptosis-related proteins (including Caspase 3 and 9, PARP-1, Bax, Bcl-2 and Survivin) after treated for 24 h, 48 h, 72 h in T98G and LN18 glioma cells. Our results (Fig. 2D) showed that after treated with PTE at different times, the apoptosis-related proteins such as cleaved caspase-3, cleaved caspase-9, cleaved PARP-1, and Bax increase significantly, while anti-apoptotic proteins Bcl-2 and Survivin reduced obviously, which further confirmed PTE induced glioma cell apoptosis may occur through the mitochondrial pathway with time-dependent characteristics.

Furthermore, we assessed whether PTE-induced cell death was caused by a caspase-dependent pathway. T98G and LN18 cells were pretreated with pan-caspase-inhibitor Z-VAD-FMK for 2 h followed by MTT, flow cytometry, and western blotting to detect cell proliferation, apoptosis, and related apoptotic proteins after 48 h of PTE. Our results indicated that caspase inhibitor consistently attenuated PTE-induced inhibition of cell growth and apoptosis, consistent with our previous research (Fig. 2E,G,H). In addition, caspase inhibitor significantly reversed the increase in pro-apoptotic proteins and decrease in anti-apoptotic proteins induced by PTE (Fig. 2F). Based on these experimental results, our discoveries demonstrated that PTE efficiently induced glioma cell apoptosis via caspase-based mitochondrial apoptosis.

### Pterostilbene provoked cell cycle arrest in the S phase

As both apoptosis and the cell cycle are important for cell proliferation, we assessed the cell cycle distribution of glioma cells (T98G, LN18, U87, and LN229) by flow cytometry following PTE treatment at various concentrations for 48 h. The results shown in Fig. 3A,B revealed that glioma cells treated with PTE, the percentage in the S-phase of the cell cycle significantly increased in a dose-dependent manner, while the percentage changes of cells in the G_1_ and G_2_/M phases were unstable and irregular. To further support these results, we detected the expression of cell-cycle-regulated proteins by western blotting. As shown in Fig. 3E, PTE led to a dose-dependent increase in phospho-histone H2A.X and Chk2 protein levels in glioma cells. Cdc25A is a key convergence point for the DNA damage checkpoint; thus, exploring the complex network between Cdc25A and CDK activity is critical. We assessed the protein levels of cdc25A, CDK2, and Cyclin A2 and found that the levels of these three proteins were reduced in glioma cells treated with PTE. These results suggested that the anti-proliferative effect of PTE in glioma cells appeared to occur by inducing S-phase arrest.

### Pterostilbene inhibited the migration, invasion and epithelial-mesenchymal transition (EMT) of glioma cells

**Figure 4:**
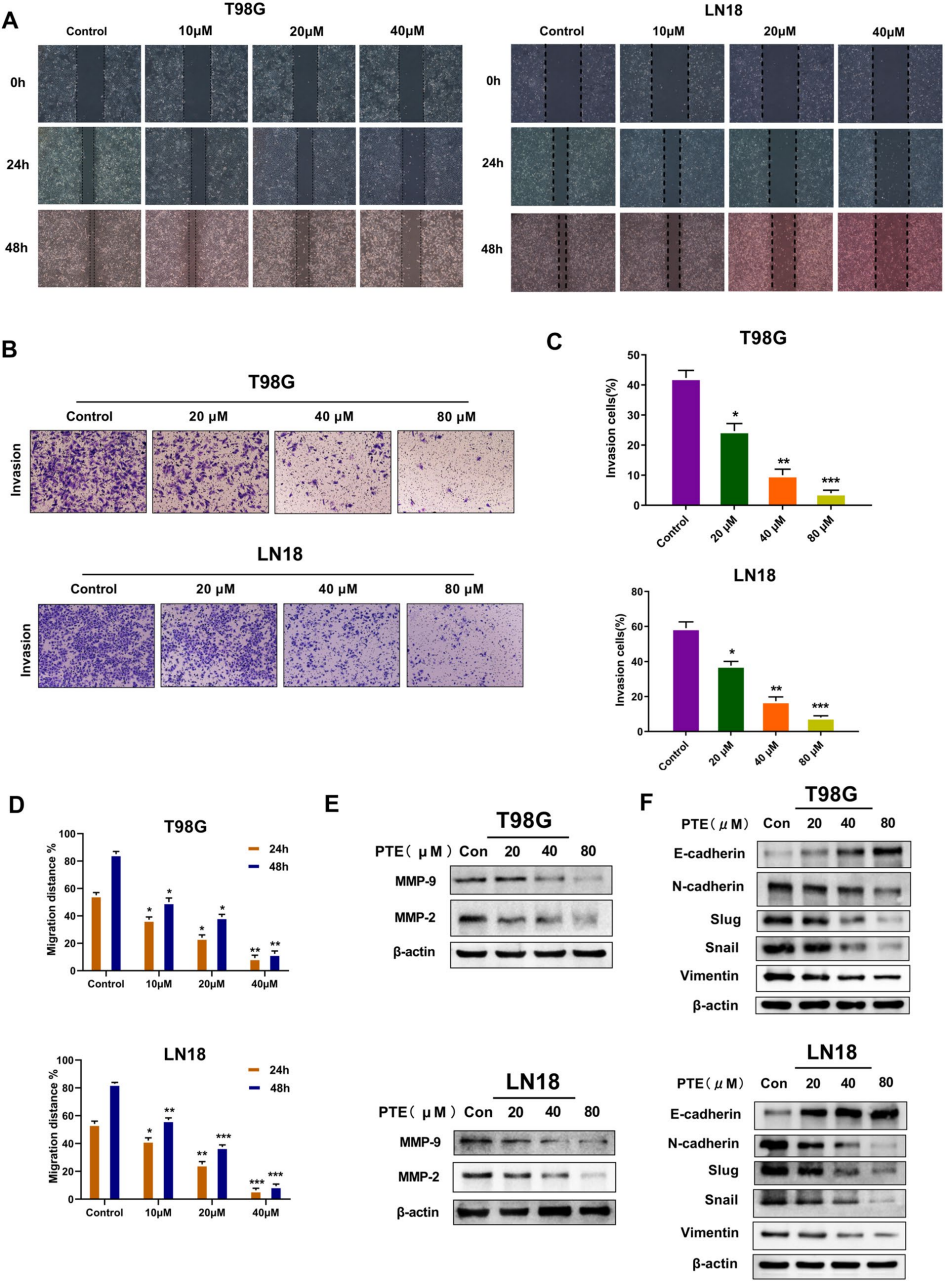
Pterostilbene inhibits the migration and invasion of glioma cells. (**A**) and (**D**) The wound-healing assay was used to detect the migration ability of T98G and LN18 cells. Representative wound healing images show migration ability of T98G and LN18 treated with PTE for 24 and 48 h. Quantification histograms showed the mean level of migration distance observed in three random fields for each condition. (**B**) and (**C**) The invasive ability of T98G and LN-18 cells was detected by the method of matrigel-coated transwell invasion assay. Representative transwell photos showed the invasion ability of T98G and LN18 cells after 48 h of exposure to PTE. Quantitative histograms showed the mean level of the numbers of cells counted in five random fields on each filter for each condition. (**E**) Western blots results showed the expression level of MMP-9 and MMP-2 proteins in T98G and LN18 cells following treatment with PTE (0, 20, 40 and 80 µM) for 48 h. (**F**) Western blot analysis was used to measure the protein levels of EMT biomarkers (E-cadherin, N-cadherin ,Slug, Snail and Vimentin) in T98G and LN18 cells treated with PTE for 48 h.β-actin was used as a loading control. Data are presented as the mean ± standard deviation (SD); n = 3; *p < 0.05, **p < 0.01 and ***p < 0.001, compared with the vehicle control group.

After studying the effects of PTE on glioma cell apoptosis and proliferation in vitro, we further investigated its ability to inhibit metastasis of glioma cells. Wound-healing assays confirmed that PTE inhibited T98G and LN18 cell migration in a dose-dependent manner (Fig. 4A,D). Similar results were also observed in the corresponding Matrigel assays that determined the invasion abilities of T98G and LN18 cells (Fig. 4B,C). Immunoblotting analysis showed that after 48 h of treatment with PTE, the expression levels of MMP-2 and MMP-9 proteins in glioma cells were significantly reduced and dose-dependent (Fig. 4E). In order to determine whether EMT is related to PTE's inhibition of glioma cell invasion, we used Western blotting to detect EMT-related markers. After 48 h of PTE treatment in T98G and LN18 cells, the expression of E-cadherin increased significantly, while the expression of N-cadherin, Slug, Snail and vimentin decreased significantly (Fig. 4F). Overall, the present data indicated that PTE may inhibit the migration and invasion of glioma cells via downregulation of MMP-2 and MMP-9 and EMT suppression.

### Pterostilbene induced the loss of mitochondrial membrane potential and production of reactive oxygen species (ROS)

**Figure 5:**
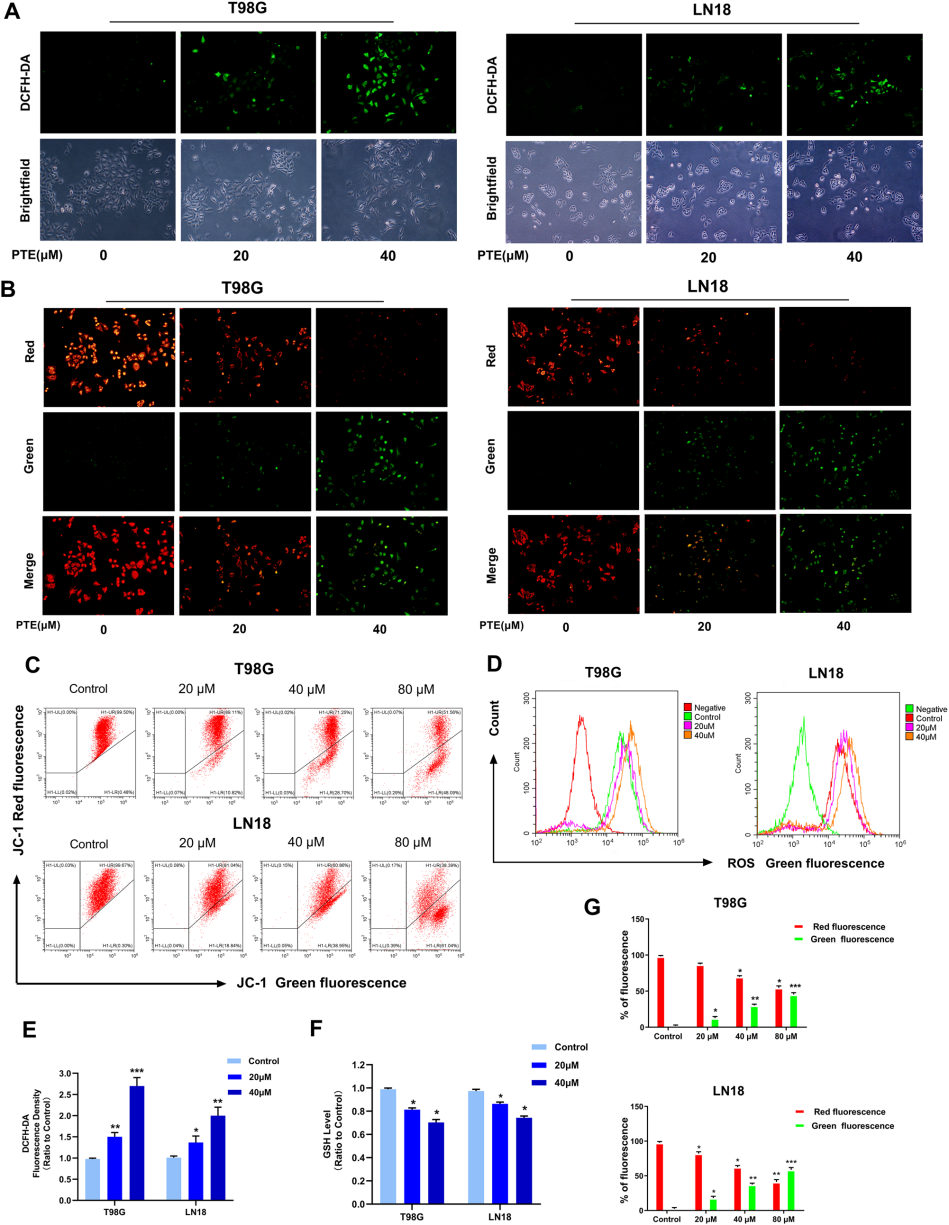
Pterostilbene induced overproduction of intracellular ROS in glioma cells. (**A**,**D**) T98G and LN18 cells were treated with PTE for 24 h. Cells were stained with 20 µM DCFH-DA at 37 °C in the dark for 20 min, and the ROS level was determined by fluorescence microscopy (10 ×) and flow cytometry. (**B**) Representative images of T98G and LN18 cells stained with JC-1 after treatment with PTE (20 and 40 µM) for 48 h, respectively, under a fluorescence microscope and a brightfield microscope (10 ×). (**C**,**G**) T98G and LN18 cells were treated with PTE for 48 h and the MMP was measured with the fluorescent mitochondrial probe JC-1 and analyzed by flow cytometry; Histogram showed the percentage of green positive cells and red negative cells in control and PTE treatment groups. (**E**) T98G and LN18 cells were treated with PTE for 24 h. Statistics analysis of the fluorescence intensity in the cells stained with DCFH-DA, which was read from a fluorescence spectrometer. (**F**) T98G and LN18 cells were treated with PTE for 24 h. Intracellular GSH level was measured by using the DTNB-GSSH reductase recycling assay kit. The GSH contents of cells were expressed as a ratio relative to the absorbance value of the control cells. Data are presented as the mean ± standard deviation (SD); n = 3; *p < 0.05, **p < 0.01 and ***p < 0.001, compared with the vehicle control group.

The breakdown of mitochondrial structural integrity is an early sign in the induction of apoptosis by intrinsic pathways^38^. To illuminate whether PTE-induced apoptosis is related to mitochondrial depolarization, we used the JC-1 MMP detection kit to assess mitochondrial membrane potential (MMP). In normal non-apoptotic cells, the JC-1 dye usually aggregates in the mitochondrial matrix and exhibits strong red fluorescence. In abnormal or apoptotic cells, JC-1 cannot accumulate in mitochondria due to the loss of mitochondrial cell membrane potential, and can only appear as a monomer in the cytoplasm and exhibit green fluorescence. As shown in Fig. 5B,C,G, increasing PTE treatment gradually enhanced the green fluorescence of cytoplasm in a dose-dependent manner, indicating that PTE interfered with and destroyed MMP, thereby activating the intrinsic apoptosis pathway. ROS is the main mediator of the ROS-dependent MMP apoptosis pathway^38^. To further evaluate whether PTE induced intracellular oxidation, we used the specific oxidation-sensitive fluorescent dye DCFH-DA to detect ROS levels in T98G and LN18 cells. As shown in Fig. 5A,D, in PTE-treated glioma cells, intracellular ROS is overproduced in a dose-dependent manner. Fluorescence microscopy with DCFH-DA staining revealed that the green fluorescence of T98G and LN18 cells treated with PTE for 24 h was markedly brighter than of the control group, presenting in a dose-dependent manner (Fig. 5E). We also investigated levels of the intracellular ROS scavenger GSH. As shown in Fig. 5F, PTE resulted in significantly decreased GSH levels in glioma cells in a dose-dependent manner. Taken together, these discoveries further support our hypothesis that PTE-induced apoptosis is mediated by increasing the levels of ROS and decreasing GSH in glioma cells.

### ROS were necessary for PTE-induced apoptosis in glioma cells

**Figure 6:**
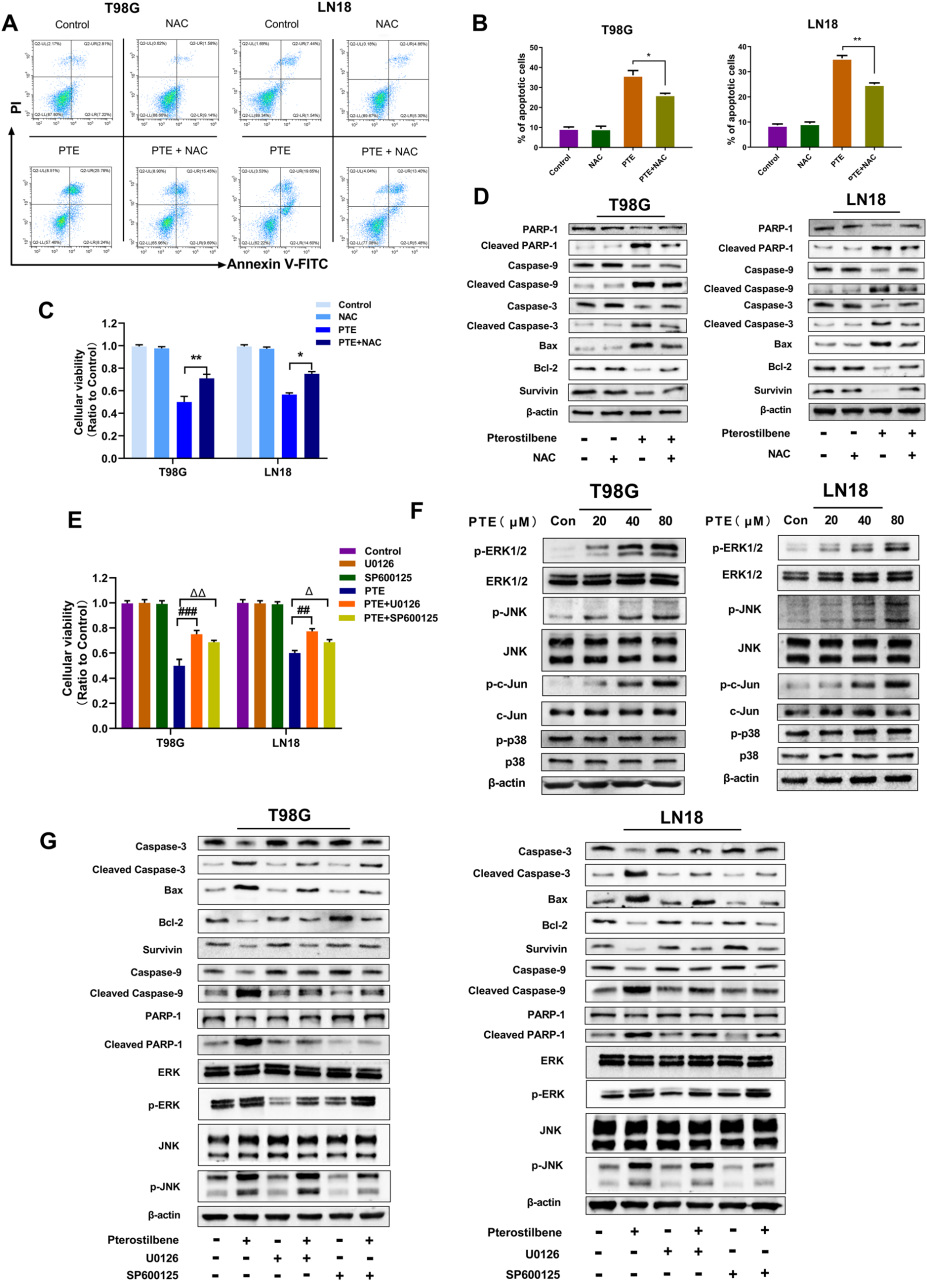
Effect of PTE on mitogen-activated protein kinase (MAPK) signaling pathway. (**A**) and (**B**) T98G and LN18 were pretreated with NAC (3 mM) for 2 h, and then treated with 40 µM PTE for 48 h. Early and late apoptotic cells were analyzed by flow cytometry. Histograms showed the mean percentage of cells in apoptosis. (**C**) The viabilities of T98G and LN18 cells that pre-incubated with antioxidant NAC (3 mM) for 2 h before PTE treated for 48 h were detected by MTT assay. (**D**) Western blotting was performed to analyze the expression levels of caspase-3, cleaved-caspase-3, caspase-9, cleaved-caspase-9, PARP-1, cleaved- PARP-1, Bax, Bcl-2 and Survivin in T98G and LN18 after PTE and PTE + NAC treatments. (**E**) U0126, SP600125 (10 µM each) were added to T98G and LN18 cells 3 h before PTE treatment for 48 h. Cell viability was determined by MTT assay. (**F**) T98G and LN18 cells were treated with PTE (20, 40, and 80 µM) for 48 h and the levels of extracellular regulated protein kinases (ERK) 1/2, phospho-ERK1/2, p38 MAPK, phospho-p38 MAPK, c-Jun N-terminal kinase (JNK), phospho-JNK, c-Jun and phospho-c-Jun were assessed by Western blot. (**G**) Western blotting was performed to analyze the apoptosis-related proteins expressions of caspase-3, cleaved-caspase-3, caspase-9, cleaved-caspase-9, PARP-1, cleaved- PARP-1, Bax, Bcl-2 ,Survivin, p-ERK, ERK, p-JNK,JNK in T98G and LN18 cells pretreated with or without U0126, SP600125 (10 µM each) for 3 h before PTE treatment for 48 h. β-actin was used as a loading control. Data are presented as the mean ± standard deviation (SD); n = 3; (*p < 0.05, **p < 0.01, PTE + NAC vs PTE); (^##^*p* < 0.01, ^###^*p* < 0.001, PTE + U0126 vs PTE); (^△^*p* < 0.05 and ^△△^*p* < 0.01, PTE + SP600125 vs PTE).

To estimate the role of ROS in PTE-induced apoptosis of glioma cells, the antioxidant NAC was applied to T98G and LN18 cells. After pretreatment with NAC (3 mmol/L), NAC reversed not only the PTE-induced reduction of cellular viability but also the increase of apoptosis in T98G and LN18 cells (Fig. 6A,B,C). As shown in Fig. 6D, NAC reversed the PTE-induced downregulation of anti-apoptotic proteins Bcl-2 and Survivin, as well as the upregulation of cleavage in the pro-apoptotic proteins Bax, cleaved caspase-3, cleaved caspase-9, and cleaved PARP-1. Therefore, these results suggested that ROS contributes to the PTE-induced apoptosis of glioma cells.

### ERK1/2 and JNK were modulated by pterostilbene-induced apoptosis

To further investigate the molecular mechanism of PTE’s antitumor effects on glioma cells, we analyzed several possible pathways by western blotting. As shown in Fig. 6F, PTE activated the phosphorylated extracellular regulated protein kinase (ERK) 1/2 and phosphorylated c-Jun n-terminal kinase (JNK) pathways and promoted their protein expression levels after treatment with PTE for 48 h. We also found that the expression of phosphorylated c-Jun, a downstream protein of JNK, was increased, while the expression of the phosphorylated p38 kinase, involved in the mitogen-activated protein kinase (MAPK) pathway, remained unchanged. To further validate the role of ERK1/2 and JNK in promoting glioma apoptosis, T98G and LN18 cells were pretreated with U0126 (a MEK inhibitor) or SP600125 (a JNK inhibitor) for 2 h and then after treated with PTE for 48 h, cell proliferation and apoptosis were tested by MTT and related apoptotic proteins were assessed by western blotting.MEK is a dual-specific protein kinase that plays a role in the mitogen-activated protein kinase cascade that controls cell growth and differentiation. MEK activates p44 and p42 MAP kinases (ERK1/2) by phosphorylating kinase subdomain VIII to activate threonine and tyrosine residues in the loop^39^. Therefore, U0126 as a MEK inhibitor,after inhibiting MEK, the downstream ERK will also be inhibited.Our data showed that MEK inhibitor (U0126) and JNK inhibitor (SP600125) attenuated the inhibition of cell growth by PTE, which is consistent with our previous studies (Fig. 6E). As can be seen in Fig. 6G, ERK1/2 inhibitor and JNK inhibitor significantly reversed the increase in cleavage of the pro-apoptotic proteins Bax, cleaved caspase-3, cleaved caspase-9, and cleaved PARP-1 and the decrease in anti-apoptotic proteins Bcl-2 and Survivin induced by PTE. We also found that when the MEK inhibitor U0126 was added for pretreatment for 3 h and then treated with PTE for 48 h, the expression of phosphorylated p-ERK reduced, and the expression of total ERK, p-JNK and total JNK did not change significantly. However, after adding the JNK inhibitor SP600125, the expression of phosphorylated p-JNK decreased, and the expression of total JNK, p-ERK and total ERK did not change significantly. From the above experimental results, we speculated that the activation of ERK1/2 and JNK plays an important role in PTE-induced glioma cell apoptosis.

### Pterostilbene inhibited the growth of glioma in vivo

**Figure 7:**
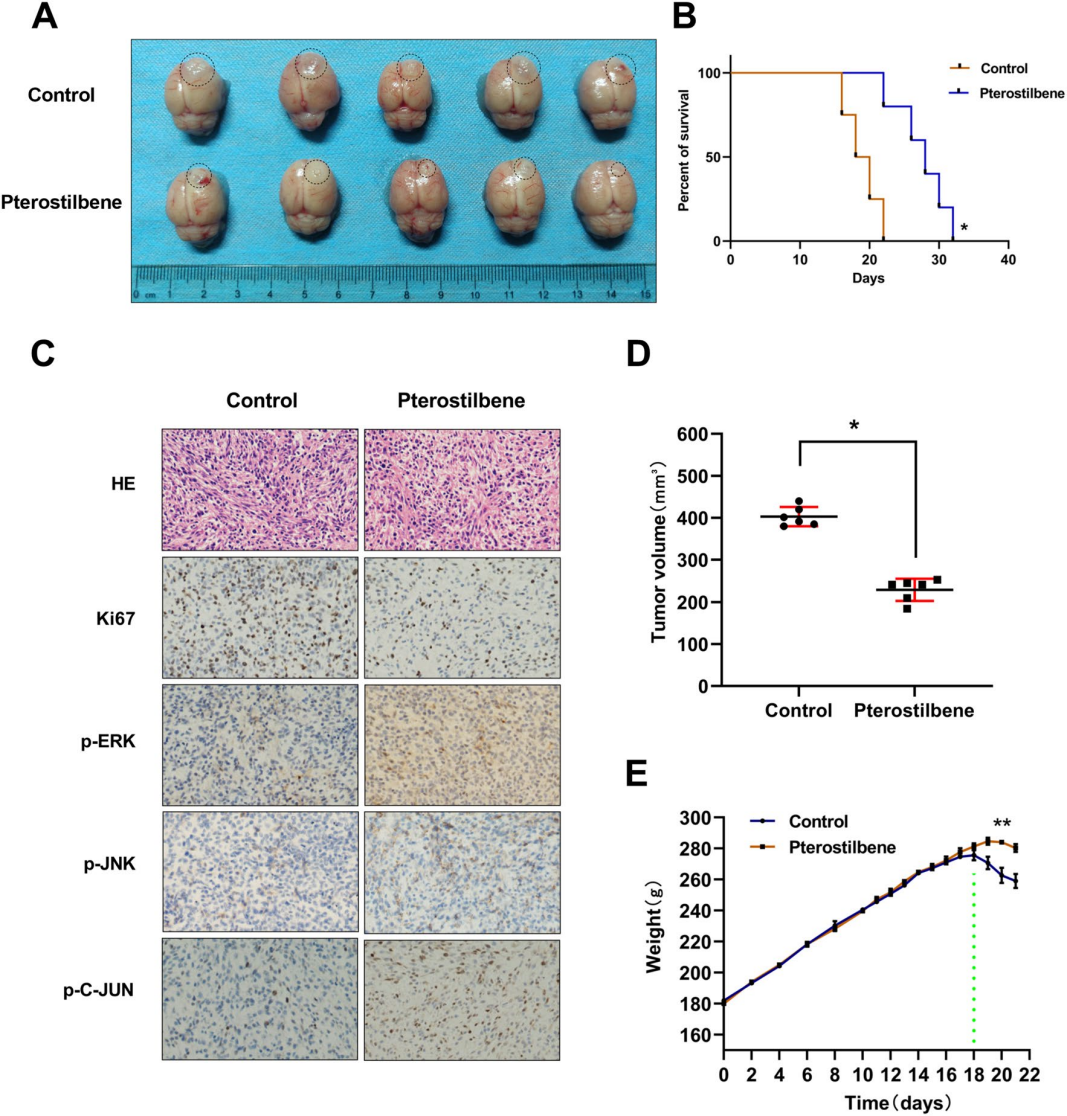
Pterostilbene inhibited the growth of glioma in vivo. (**A**) Tumor samples were collected and photographs show tumor volume in different groups. (**B**) Survival of brain tumor bearing rats were recorded and represented in a Kaplan–Meier plot. (**C**) The intracranial tumor tissues were stained with HE and immunohistochemistry analysis using anti-Ki-67, anti-p-ERK1/2, p-JNK, p–C-JUN antibodies. Positive staining of the cells is brown. (**D**) Tumor volumes in control groups and PTE treatment groups. The tumor volume was calculated using formula: 0.5 × A × B^2^, which A was the length of the tumor and B was the width. (**E**) Rat body weights were measured for 21 days. Data are presented as the mean ± standard deviation (SD); n = 3; *p < 0.05, **p < 0.01, compared with the vehicle control group.

**Figure 8:**
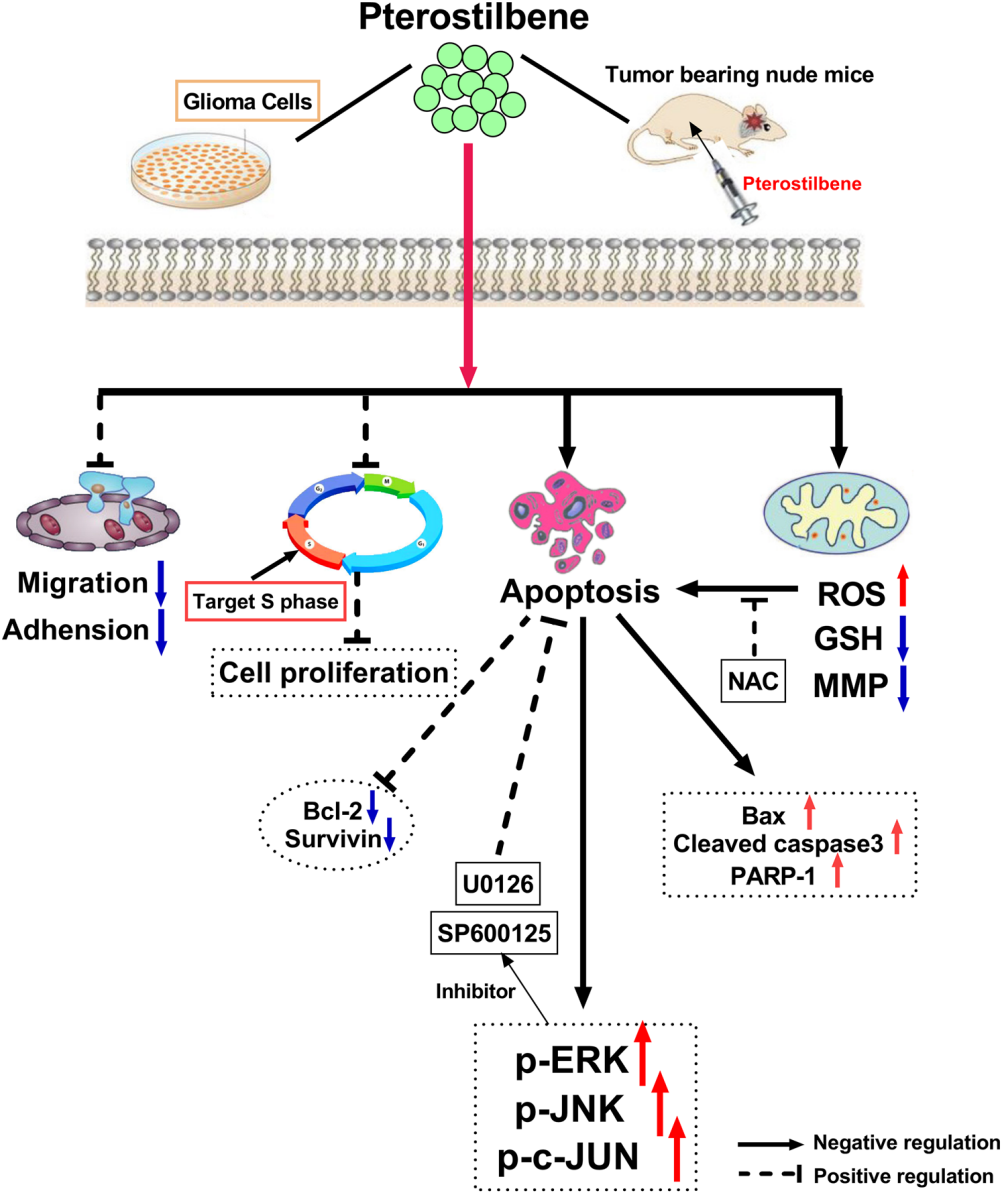
A possible network, describes the main mechanisms of PTE treatment.

We further validate the antitumor effects of PTE in a Wistar rat model with intracranial orthotopic xenograft glioma using C6 cells. Seven days after tumor induction, rats were randomly divided into 2 groups: the control group (n = 6) and the PTE group (n = 6) (50 mg/kg). The control group was treated with 5% dimethyl sulfoxide (DMSO) and saline and the PTE group was treated with PTE for 14 days via intraperitoneal injection. As shown in Fig. 7B, PTE significantly prolonged the median survival time of tumor-bearing rats. At the same time, the volume of the transplanted tumor in the PTE group was significantly smaller than that in the 5% DMSO and saline treated group (Fig. 7A,D). At the same time, we measured rat body weights daily and the results showed that there was no significant difference in body weight changes between the groups within 14 days (Fig. 7E). After 14 days, weight gain in the PTE-treated group slowed, while the control group showed weight loss (Fig. 7E). Tumor area was visualized by H&E staining; tumors treated with pterostilbene had significantly decreased Ki-67 levels (Fig. 7C). Moreover, IHC analysis revealed that pterostilbene enhanced the expression of p-ERK1/2, p-JNK, and p–c-Jun in the C6 xenograft-bearing rats (Fig. 7C). These results indicated that pterostilbene inhibited the growth of glioma in vivo and extended survival in these in-vivo glioma models. Figure 8 presents a possible network describing the main mechanisms influenced by PTE.

## Discussion

Although the early stages of GBM can be managed by surgery and several therapeutic agents, the inherent resistance to chemotherapy and radiation of GBM is an important factor leading to aggressive clinical courses and poor prognosis^40^. In order to improve the current state of unsatisfactory outcomes, the evaluation of development of new chemo-preventive and/or chemo-therapeutic agents is an essential and challenging task.

Recent studies have shown that pterostilbene(PTE)—a dimethyl analog of resveratrol—when compared to resveratrol, has an approximately fourfold greater (about 80% versus 20%) oral bioavailability and 7.5-fold (105 min vs 14 min) longer half-life^41,42^. Meanwhile, a prospective, randomized, double-blind, placebo-controlled clinical trial showed that pterostilbene is well tolerated and safe in clinical patients^43^. In addition, the antitumor effect of pterostilbene involves a variety of molecules and signaling pathways. For example, Yu et al. found that pterostilbene induced human hepatocellular carcinoma cell death in an endoplasmic reticulum stress and autophagy-dependent manner through the phospho-eukaryotic initiation factor 2α/activating transcription factor-4/LC3 pathway^44^. However, prior to this study, the effects of pterostilbene on glioma cells and its mechanism have not yet been elucidated.

In the present study, we evaluated the effects of PTE on glioma cells and further explored its potential molecular mechanism. First, the outcomes of our in vitro experiments showed that PTE significantly inhibited the proliferation of GBM cells, including T98G, LN18, U87, U229, and C6 cells, in a time and dose-dependent manner; furthermore, colony formation outcomes were consistent with this result. Meanwhile, our results demonstrated that PTE has little effect of HUVECs. We also found that the sensitivity of glioma cells to pterostilbene is prone to significant variability in IC_50_ values (ranging from 22.30 to 46.18 μM); this may stem from the different origins and protein expression profiles of these cell lines^45^.

Tumor progression includes proliferation, migration, invasion, adhesion, and death. Cancer cell migration and invasion are key steps for tumor metastasis and inhibition of these steps is an important approach for anti-tumor treatments^46^. Invasive tumor cells must degrade the extracellular matrix through metalloproteinases (MMPs), then change the connection and adhesion between cells. Our results demonstrated that the migration and invasion of T98G and LN18 cells were markedly decreased by PTE in a dose-dependent manner. It has been reported that MMP-2 and MMP-9 are extracellular proteolytic enzymes that play an important role in tumor invasion^47^. In this study, we confirmed that PTE significantly inhibited the expression of MMP-2 and MMP-9 proteins, consistent with the above theory. Studies have shown that tumor cells usually show EMT characteristics in tumor invasion and metastasis^48^. The characteristic of EMT is that epithelial cells obtaining mesenchymal behavior due to the loss of intercellular adhesion and apicobasal polarity, and acquire mesenchymal proteins, thereby promoting the proliferation of cancer cells^49,50^. The data of immunoblotting experiments in this study showed that after treated for 48 h of PTE, the expression of E-cadherin increased, while the expression of the expression of N-cadherin, Slug, Snail and vimentin decreased significantly. The current results indicate PTE may inhibit the migration and invasion of glioma cells via downregulation of MMP-2 and MMP-9 accompanied by EMT progression.

As we known, apoptosis and cell cycle arrest can induce the inhibition of cell proliferation. In the current study, some hallmarks of apoptosis were tested in glioma cells treated with PTE. Based on flow cytometry results, PTE significantly induced glioma cell apoptosis in a dose-dependent manner, especially early apoptosis. Moreover, we used Hoechst 33342 staining to observe the effect of PTE on the morphological changes in glioma cells and found that it revealed characteristics of apoptosis, including apparent nuclear condensation, nuclear fragmentation, and enhanced blue brightness. Apoptosis occurs mainly through an extrinsic death receptor-mediated pathway (activated by caspase-8) or intrinsic mitochondria-mediated pathway (activated by caspase-9 and caspase-3)^51^. Caspase-3 is considered to be the most important regulator of apoptosis, while caspase-9 is considered to be the main regulator of mitochondrial-mediated apoptosis^52^. The intrinsic mitochondria-mediated pathway is characterized by an imbalance in the expression levels of Bcl-2 family proteins and the depolarization of mitochondrial membrane potential (MMP)^53^. MMP is an indicator of mitochondrial membrane permeability, which is decreased in early apoptosis leading to the activation of caspase-9^54^. Mitochondria have been proposed as new targets for cancer intervention and treatment^55^. In our study, we found that T98G and LN18 cells treated with PTE showed a significant loss of mitochondrial membrane potential as well as Bcl-2 family dysregulation. Our western blot analysis showed that PTE led to significantly increases in the protein expression levels of cleaved-caspase-3, cleaved-caspase-9, BAX, and cleaved-PARP-1 and decreases in the levels of Bcl-2 and Survivin dose-dependently. These results indicated that PTE induced apoptosis in glioma cells through intrinsic apoptotic pathways, as reflected by the loss of MMP and the disorder of Bcl-2 family expression levels. To further investigate whether the activation of the caspase cascades is indispensable for PTE-induced apoptosis, we added the pan-caspase inhibitor Z-VAD-FMK to T98G and LN18 cells. MTT, Western blot and flow cytometry results showed that pretreatment with pan-caspase inhibitor Z-VAD-FMK significantly reversed PTE-induced glioma cell death, suggesting that PTE induced cell apoptosis through an intrinsic caspase-dependent mechanism. However, Tolomeo et al. found that the pan-caspase inhibitor Z-VAD-FMK did not reverse PTE-induced apoptosis in leukemia cells, suggesting that PTE may work through a caspase-independent pathway^56^. Therefore, a mechanism may exist that is independent of the caspase apoptotic pathway or a modality of cellular death for PTE-mediated cell death.

Oxidative stress results from imbalances caused by excess reactive oxygen species (ROS)^57^. ROS play a vital role in inducing apoptosis in cancer cells^58^. Meanwhile, ROS is the main mediator of the ROS-dependent mitochondria-mediated apoptosis pathway^38^. Previous studies have shown that PTE induces malignant tumor cell apoptosis by affecting the production of ROS, including in non-small-cell lung cancer (NSCLC)^20^, T-cell leukemia/lymphoma^26^, multiple myeloma^59^, human acute myeloid leukemia cells^27^, and breast cancer cells^60^. Therefore, inducing apoptosis in tumor cells by disrupting balance in the antioxidant system and promoting the accumulation of intracellular ROS is an important mechanism in research on antitumor therapies^8,61^. In the present study, lower concentrations of PTE (20 μM) significantly increased ROS accumulation and reduced GSH in a dose-dependent manner, confirming the important role of ROS as an early event in apoptotic signaling. As an antioxidant, NAC enhances tissue-specific antioxidant activity and is often used to identify the role of reactive oxygen species in various biological reactions. In the current study, we confirmed that pretreatment with antioxidant NAC could reverse the inhibitory effects of PTE on proliferation and apoptosis in glioma cells. Therefore, we demonstrated that ROS played a decisive role in PTE-induced mitochondrial apoptosis.

The most important feature of tumors is infinite proliferation—i.e., immortalization. This immortalization of tumor cells can cause cell cycle disorders and uncontrolled cell proliferation^62^. Therefore, the cell cycle is also an important target to be monitored in anti-tumor therapies. Previous research showed that S-phase checkpoint detected DNA damage and repair. H2A.X is a variant of the histone H2A histone family. Phosphorylated H2A.X plays a crucial role in the DNA damage response and is the key to DNA repair protein assembly during cell cycle progression^63^. According to our flow cytometry results, glioma cells were arrested in the S phase after 48 h of incubation with different concentrations of PTE, which is consistent with previous research by Alosi et al.^60^. In addition, western blot analysis validated that PTE induced an increase in the protein levels of phospho-H2A.X. Additional results showed that augmented expression levels of CHK2—a protein kinase that is a crucial mediator of DNA damage checkpoints—were observed under PTE treatment. It phosphorylates lots of proteins involved in cell cycle control, including cdc25A^64^. We also found that PTE downregulated protein levels of cyclin A2, CDK2, and cdc25A. These results further confirm that CHK2 expression is triggered by PTE-induced DNA damage and cdc25A expression. We have fully confirmed the DNA damage caused by PTE by increasing the expression of H2A.X and CHK2, which also provides new ideas for future investigations of its molecular mechanism.

The MAPK signaling pathway plays a vital role in regulating cell proliferation, differentiation, migration, and apoptosis in various types of cancers. MAPKs are comprised of a family of protein kinases, including ERK1/2, p38, and JNK^65^. Studies have shown that the induction of apoptosis in certain tumor cells is related to the MAPK signal transduction pathway^10,66^. In the present study, we studied MAPK-related protein levels upon treatment with different concentrations of PTE (20, 40, 80 μM) in glioma cells. Our results showed that PTE did not change the total levels of ERK1/2, JNK, and P38 in glioma cells while significantly increasing the phosphorylation levels of ERK1/2 and JNK, but had no significant effects on phosphorylation levels of p38. Our analysis of the JNK/c-Jun pathway uncovered that PTE also increased the phosphorylation of c-Jun. In addition, in order to further explore the correlation between ERK1/2 and JNK with PTE-induced glioma cell apoptosis, we pretreated with the specific MEK inhibitor U0126 and JNK inhibitor SP600125; the MTT and western blotting results showed that specific JNK and MEK inhibitors reversed the inhibition of PTE on glioma cell proliferation and PTE-induced glioma apoptosis. Taken together, our discoveries validate that the ERK/JNK signaling pathways play a key role in the apoptosis of glioma cells triggered by PTE through a caspase-dependent mechanism.

In order to verify the anti-tumor effect of PTE in vivo, we established a xenograft animal model. Due to its greater lipophilicity, PTE exhibits higher bioavailability and is more likely to cross the blood–brain barrier (BBB); it may therefore be more resultful in central nervous system diseases^67^. Previous studies showed that the natural compound PTE could penetrate the BBB and significantly reduce the growth of metastatic tumors in the brains of breast cancer patients by inhibiting the expression of c-Met^68^. In this study, tumor-bearing rats were injected intraperitoneally with 5% DMSO and normal saline or 50 mg/kg PTE for two weeks. The results showed that PTE treatment significantly reduced tumor volume and prolonged median survival. In addition, no differences in rat body weight were observed between the treatment groups, indicating that PTE may be well tolerated in vivo. Further, our immunohistochemical results showed that PTE promoted the expression levels of p-ERK, p-JNK, and p–C-JUN and inhibited the expression level of Ki67, confirming the role of the MAPK signaling pathway in the antitumor effect of PTE.

In conclusion, our study clarifies that PTE is an effective antitumor agent for glioma cells in vivo and in vitro through inhibiting cell viability, inducing S-phase arrest of the cell cycle, and triggering mitochondria-mediated apoptosis. Regarding its mechanism, caspase-dependent apoptosis of glioma cells by PTE is connected with to mitochondrial dysfunction and excessive ROS production. MAPKs (ERK1/2 and JNK) played a vital role in the chemo-preventive effect of this polyphenolic compound in glioma cells. In addition, our rat xenograft model demonstrated that intravenous injection of PTE can obviously inhibit tumor growth. Therefore, because of its higher bioavailability and ease of crossing the BBB, PTE offers a potential novel treatment for GBM patients.

However, our experiments failed to measure the concentration of PTE in brain tissue to assess the blood–brain barrier penetration and the toxicity of PTE to various organs of the body. These research limitations will be further investigated in the future.

## Materials and methods

### Cell culture

Human T98G, LN18, LN229, U87, and rat C6 glioma cells were purchased from the American Type Culture Collection (ATCC; Manassas, VA, USA). All cell lines were cultured in Dulbecco’s modified Eagle medium (DMEM) supplemented with 10% fetal bovine serum and penicillin–streptomycin (100 U/mL) in a humidified atmosphere of 5% CO_2_ at 37 °C. HUVECs (human umbilical vein epithelial cells) were obtained from the Shanghai Institute of Cell Biology, Chinese Academy of Sciences (Shanghai, China) and cultured according to the instructions provided by the source.

### Reagents

Pterostilbene (purity of > 98%) was purchased from Chengdu Croma Biological Technology Co., Ltd. (Chengdu, China) and dissolved in dimethyl-sulfoxide DMSO (Sigma-Aldrich, St. Louis, MO, USA) then stored in the dark at − 20 °C. NAC (*N*-acetyl-l-cysteine) and the JC-1 Mitochondrial Membrane Potential Detection Kit were purchased from Beyotime Biotechnology Company (Shanghai, China). U0126, SP600125, and Z-VAD-FMK were purchased from Selleck Chemicals (Houston, TX, USA); all reagents were diluted with DMEM to the desired final concentration at the time of use.

### Cell viability analysis

T98G (5 × 10^3^ cells/well), LN18 (8 × 10^3^ cells/well), U87 (5 × 10^3^ cells/well), LN229 (5 × 10^3^ cells/well), C6 (5 × 10^3^ cells/well) glioma cells and HUVECs (5 × 10^3^ cells/well) were seeded in 96-well plates and cultured for 24 h. All cells were then treated with various concentrations (0, 20, 40, 80 and 100 µM) for indicated time (24, 48, 72 h). Cell viability was evaluated using an MTT assay and the absorbance at 570 nm was determined by a microplate reader (Bio-Tek Instruments, USA).

### Colony formation assay

T98G (1 × 10^3^ cells/well) and LN18 (1 × 10^3^ cells/well) cells were seeded in 6-well plates and cultured for 8 h to attach to the plates. Then cells were treated with pterostilbene at indicated concentrations (20, 40, and 80 µM) for approximately 10 days, following cells were fixed with 4% paraformaldehyde and stained with 0.1% crystal violet. The number of colonies > 50 cells in each well was counted with the help of ImageJ (NIH, Bethesda, USA).

### Analysis of apoptosis

The Annexin-V/PI apoptosis detection kit (Becton Dickinson, San Diego, CA, USA) was used to evaluate the apoptosis of glioma cells induced by PTE according to the provided protocol. Briefly, T98G, LN18, LN229, and U87 glioma cells were treated with PTE (20, 40, and 80 µM) for 48 h. Next, collected cells were subjected to Annexin V-FITC/PI double-staining and then analyzed using a flow cytometer (Becton Dickinson, San Diego, CA, USA). The rate of cell apoptosis was analyzed using FlowJo version 10.0 software (FlowJo LLC/Becton Dickinson, Ashland, OR, USA).

### Hoechst 33342 staining

Hoechst 33342 staining was used to observe the morphological changes of PTE on glioma cells by fluorescence microscope. Briefly, cells (8 × 10^4^/mL) were seeded in 6-well plates and then treated with PTE (20, 40, and 80 µM) for 48 h. Next, after washing with PBS, the cells were stained with Hoechst 33342 (Beyotime) in the dark for 25 min at 37 °C. Cells were then observed and photographed using a fluorescence microscope (Olympus IX53/DP80, Tokyo, Japan).

### Cell cycle analysis by flow cytometry

Flow cytometry with PI/RNase staining (BD Biosciences, San Jose, CA, USA) was used to detect the effects of PTE in cell cycle distribution. Briefly, T98G, LN18, LN229, U87, and C6 glioma cells were seeded in 6-well plates and cultured 8 h. Then, the cells were treated with PTE (20, 40, and 80 µM) and harvested for 48 h. Cells were fixed with 70% ethanol overnight at − 20 °C and incubated with PI/RNase in the dark for 15 min. Stained cells were subsequently analyzed with a flow cytometer (Becton Dickinson, San Diego, CA, USA).

### Transwell and wound healing assays

24-well Matrigel-coated Transwell invasion assay (8 μm pore size, Millipore, Darmstadt, Germany) was used to assess the invasion capability of glioma cells. Suspension of cells (2 × 10^4^/200 μL) with serum-free medium and various concentrations of PTE then plated on the top side of the membrane coated with Matrigel (BD) and incubated at 37 °C for 48 h. Cells that crossed to the underside of the Transwell membrane were stained with 0.1% crystal violet, then observed and photographed under an inverted microscope (Olympus IX53/DP80).

Wound healing assays was used to monitor the migration capacity of glioma cells. Briefly, cells (2 × 10^5^ cells/well) were seeded in 6-well plates. When the cells reached 90% confluence, we used the end of a 10-μl steriled micropipette tip to make a scratch. The tip was moved vertically downward on the 6-well plate to ensure that a consistent wide scratch. Then the cells were washed 3 times with PBS and cultured with serum-free medium. Images of scratches were used to monitor the migration of cells to the cleared zone at 0, 24, and 48 h after treatment with various concentrations of PTE using a microscope equipped with a camera (Olympus IX53/DP80).

### Measurement of intracellular reactive oxygen species (ROS) and intracellular glutathione (GSH)

The redox-sensitive dye DCFH-DA (Beyotime) was used to evaluate the average levels of intracellular ROS in T98G and LN18 glioma cells. Briefly, cells were treated with PTE (20, 40 µM) for 24 h in the corresponding incubator. Then cells were harvested and stained with 20 μM DCFH-DA for 30 min in the dark. Fluorescence intensity was detected by a fluorescence spectrometer and flow cytometer (BD) and observed under an Olympus IX53/DP80 fluorescence microscope.

T98G and LN18 glioma cells were treated with indicated concentration of PTE for 24 h and then lysed. DTNB-GSSH reductase recycling assay kit (Beyotime) was used to measure the intracellular GSH levels according to the manufacturer’s instructions. GSH levels were normalized based on the protein concentrations of each sample. The content of GSH was expressed as the ratio (cultured cells / control cells) of the absorbance values.

### Measurement of mitochondrial membrane potential

Mitochondrial membrane potential was detected by MMP detection kit with JC-1 (Beyotime) according to the manufacturer's instructions. Briefly, T98G (2 × 10^5^ cells/well), LN18 (2 × 10^5^ cells/well) glioma cells were seeded in 6-well microplates, cultured 24 h and then stained with JC-1 at 37 °C in 5% CO_2_ for 30 min after treated with PTE (10, 20 µM) for 48 h. Fluorescence intensity was monitored by fluorescence microscopy (Olympus IX71) and flow cytometry (BD); mitochondrial depolarization was evaluated by measuring the decrease in the red/green fluorescence intensity ratio.

### Western blot

Glioma cells treated with various concentrations of PTE for 48 h were collected and lysed in RIPA buffer (Beyotime Biotechnology, Shanghai, China). The concentrations of the protein samples were calculated by a BCA protein assay kit following the instruction (Beyotime). Protein samples (containing 30 μg of total protein) were separated by SDS-PAGE and transferred to PVDF membranes (Merck Millipore, Burlington, MA, USA). Next, the membranes were blocked in TBS with 5% non-fat dried milk at 20 °C for 30 min. Finally, the membranes were incubated with indicated primary antibody overnight at 4 °C. Primary antibodies for Bax, Bcl-2, Survivin, MMP2, MMP9, ERK1/2, phospho-ERK1/2 (p-ERK1/2), p38, phospho-p38 (p-p38), phospho-histone H2A.X, cdc25A, CDK2, and β-actin were purchased from Cell Signaling Technology (Beverly, MA, USA). Primary antibodies for Ki-67, JNK, c-JUN, phospho-JNK (p-JNK), phospho-C-JUN (p–c-JUN), PARP-1, cleaved PARP-1, caspase-3, cleaved caspase-3, caspase-9, cleaved caspase-9 were purchased from Abcam (Cambridge, MA, USA). After incubation with secondary antibody (anti-rabbit or anti-mouse IgG) at 20 °C for 2 h, the membranes were visualized using the Gel Imaging System from Sage Creation Science Co., Ltd (Beijing, China).

### Immunohistochemistry

Immunohistochemistry was used to detect the protein expression in rat xenograft tumors. Target tumors were fixed with 4% paraformaldehyde for 24 h. Paraffin-embedded blocks were used to prepare 5 μm thick sections. After deparaffinizing, dehydrating, 3% hydrogen peroxide to block endogenous peroxidase activity, antigen repair, and conventional serum blocking, finally the sections were incubated overnight at 4 °C with specific primary antibodies, including Ki67 (1:200), p-ERK1/2 (1:400), p-JNK (1:200), and p–c-JUN (1:250) .Then sections were incubated with second antibody. The peroxidase reaction was performed using DAB counterstained with Meyer's hematoxylin. Finally, the sections were visualized and images were observed under a microscope (Olympus IX71).

### Xenograft tumor model

Male Wistar rats (weighing 160–180 g) were purchased from the Experimental Animal Center of Jilin University (Changchun, China). The study was approved by the ethics committee of the First Hospital of Jilin University. Animal experiments and animal experiment protocols were carried out in compliance with the ARRIVE 2.0 guidelines^69^. All rats were adapted to the laboratory environment before the experiment. After anesthetizing, the head of each rat was mounted into a stereotactic frame. A burr hole (1 mm diameter) was made at the right cranial bone, located at 3 mm next to the anterior fontanelle and 1 mm in front of the coronal suture. PBS (10 µL), containing 2 × 10^6^ C6 cells, was stereotactically implanted through a 10 µL microinjector to a 5 mm depth from the surface. After one week, the rats were randomly divided into two groups: control group (vehicle) and PTE (50 mg/kg, once daily, intraperitoneal injection, two weeks). Two weeks later, the brains of rat were resected, fixed with 4% paraformaldehyde and blocked by paraffin after anesthesia. The tumor volume was calculated according to the formula: 0.5 × L × W × H, where L represents the length, W represents the width and H represents the width of the tumor. Deparaffinized tumor sections were stained with hematoxylin and eosin (H&E) and incubated with specific antibodies. Detection was performed with an avidin–biotin-HRP complex and diaminobenzidine as a chromogen. Nuclei were stained with hematoxylin.

### Protocol approval

All experimental methods of this study were approved by the Research Committee of the First Hospital of Jilin University. All experiments were conducted in accordance with the guidelines issued by the committee. All animal experiments were approved by the Institutional Animal Care and Use Committee of the First Hospital of Jilin University and conformed to relevant international guidelines and regulations.

### Statistical analysis

Quantitative data were obtained from at least three independent experiments and were expressed as the means ± standard deviation (SD). Groups were compared with the unpaired Student's t-test, and multiple groups were compared by one-way ANOVA. *p* < 0.05 were considered to represent statistical significance. All the analyses were performed with GraphPad Prism 8.0 (San Diego, CA, USA) (https://www.graphpad.com/).

## Supplementary Information

Supplementary Information
