# Supplementary material for: Pterostilbene promotes mitochondrial apoptosis and inhibits proliferation in glioma cells
Source: Sci Rep. 2021 Mar 18;11:6381. doi: 10.1038/s41598-021-85908-w (PMC7973728; doi:10.1038/s41598-021-85908-w)
Supplement: Supplementary file 1 — Supplementary Information [file 41598_2021_85908_MOESM1_ESM.docx]

**Supporting information**

**Pterostilbene promotes mitochondrial apoptosis and inhibits proliferation in glioma cells**

Haijun Gao^1,2^, Ziqiang Liu^1^, Weidong Xu^1^, Qunhui Wang^1^, Chaochao Zhang^1^, Yaonan Ding^1^, Weiguang Nie^1^, Jiacheng Lai^1^, Yong Chen^1,*^, Haiyan Huang^1,*^

^1^ Department of Neurosurgery, First Hospital of Jilin University, Changchun 130021, Jilin Province, China.

^2^ Department of Neurosurgery, Fenyang Hospital of Shanxi Province, Fengyang 032200, Shanxi Province, China.

***Corresponding author：**

Haiyan Huang: Department of Neurosurgery, First Hospital of Jilin University, Changchun, 130021, China. Tel: +86 13578967878.

E-mail address: [huanghy@jlu.edu.cn](mailto:huanghy@jlu.edu.cn).

***Co-corresponding author：**

Yong Chen: Department of Neurosurgery, First Hospital of Jilin University, Changchun, China. Tel: +86 13654400697.

E-mail address: chen_yong@jlu.edu.cn

**Western blot original images Figure 2C**

**
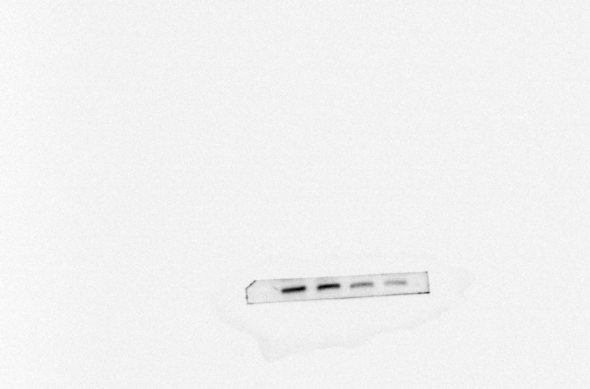

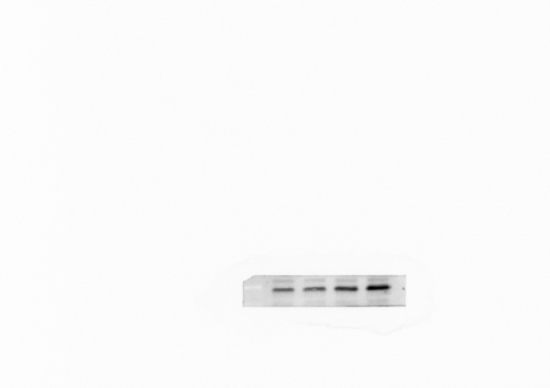
**

**Figure 2C-T98G-caspase-3 Figure 2C-T98G-Cleaved-caspase-3**

**
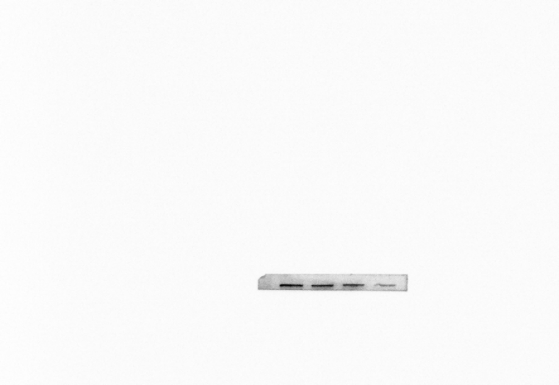

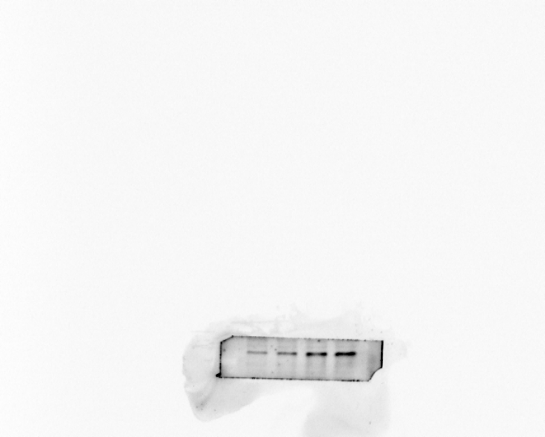
**

**Figure 2C-T98G-PARP-1 Figure 2C-T98G-Cleaved-PARP-1**

**
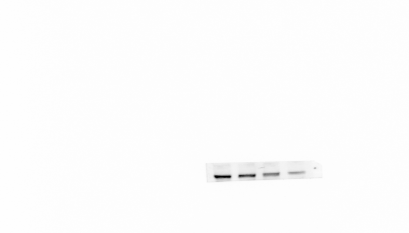

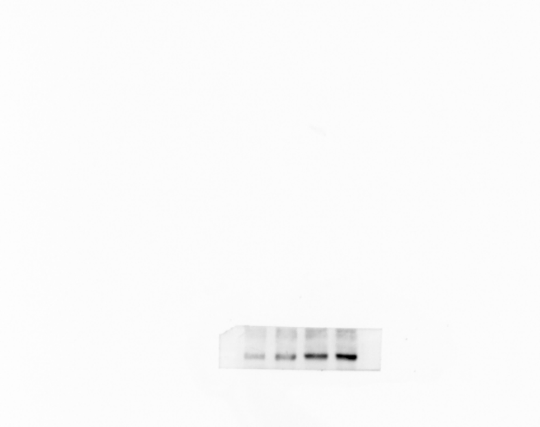
**

**Figure 2C-T98G-caspase-9 Figure 2C-T98G-Cleaved-caspase-9**

**
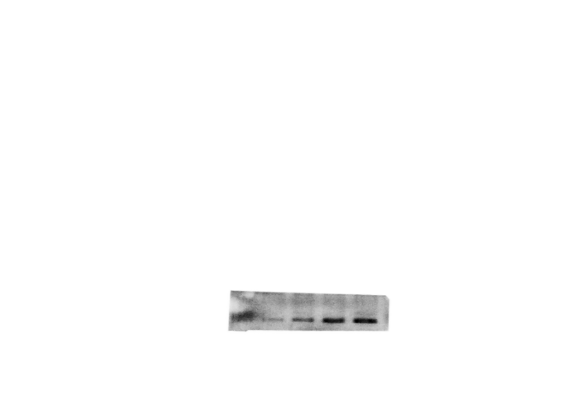

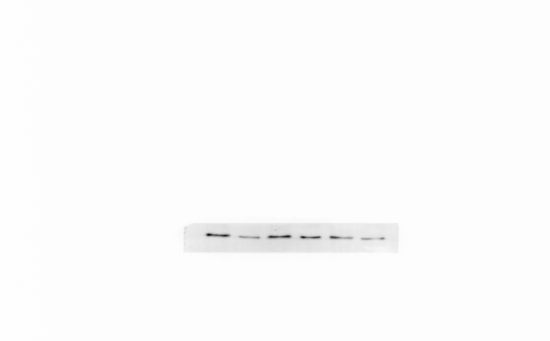
**

**Figure 2C-T98G-Bax Figure 2C-T98G-Bcl-2**

**
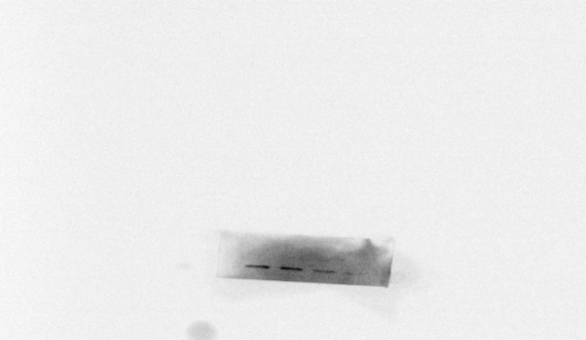

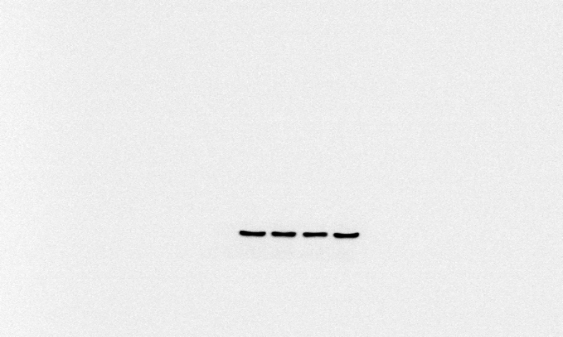
**

**Figure 2C-T98G-Survivin Figure 2C-T98G-β-actin**

**
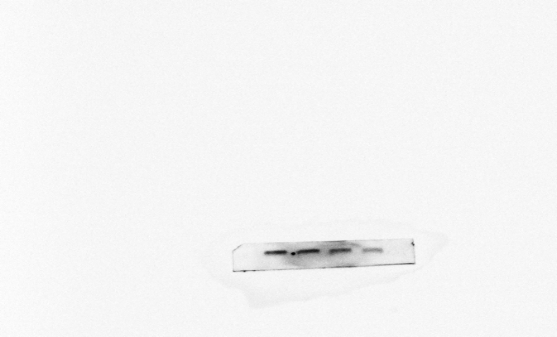

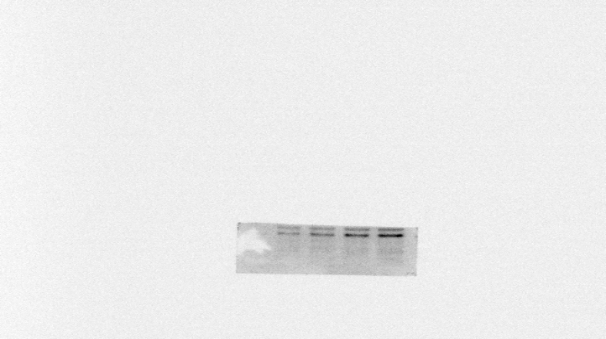
**

**Figure 2C-LN18-Caspase-3 Figure 2C-LN18-Cleaved Caspase-3**

**
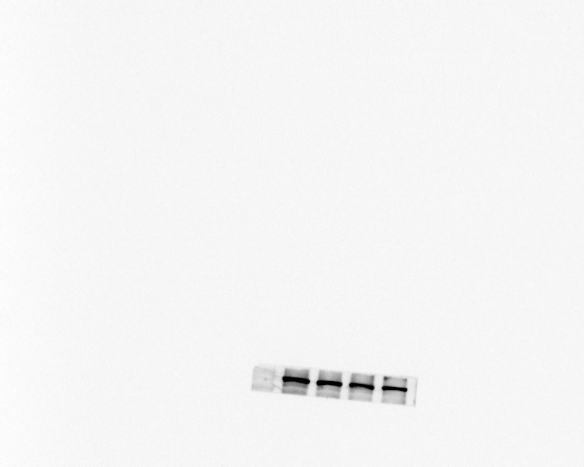

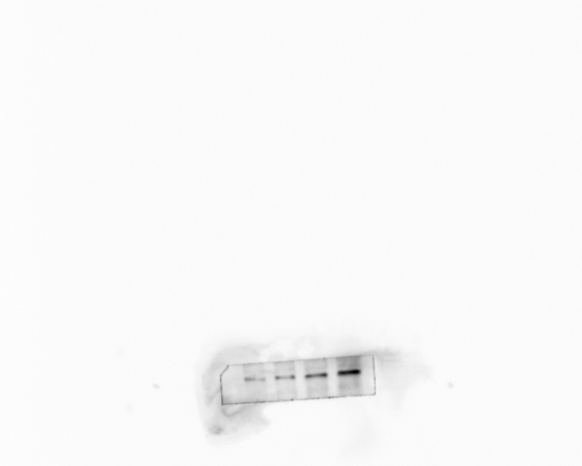
**

**Figure 2C-LN18-PARP-1 Figure 2C-LN18-Cleaved-PARP-1**

**
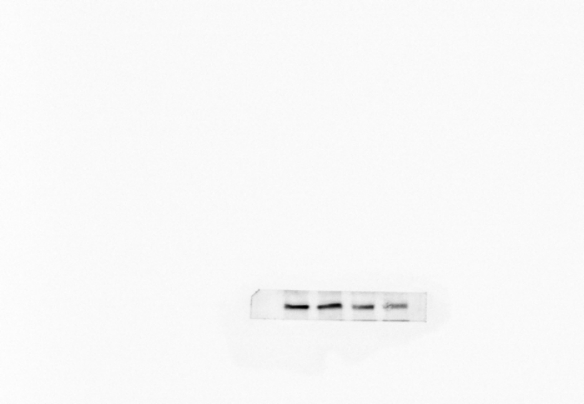

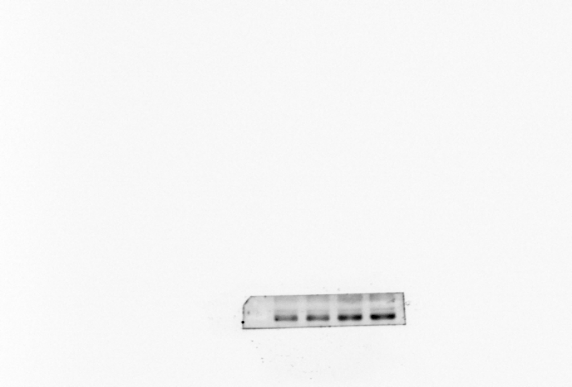
**

**Figure 2C-LN18-Caspase-9 Figure 2C-LN18-Cleaved-Caspase-9**

**
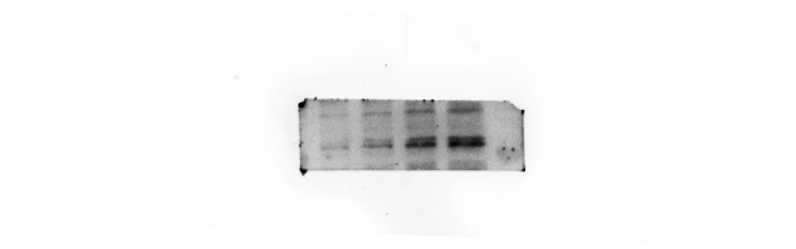

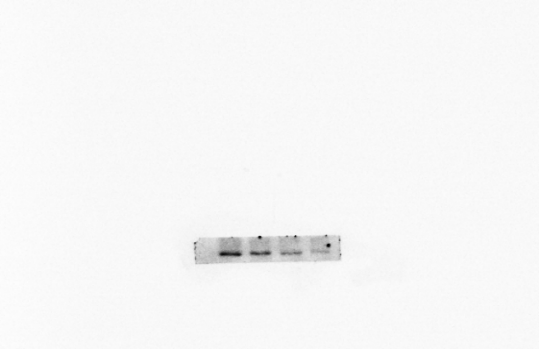
**

**Figure 2C-LN18-Bax Figure 2C-LN18-Bcl-2**

**
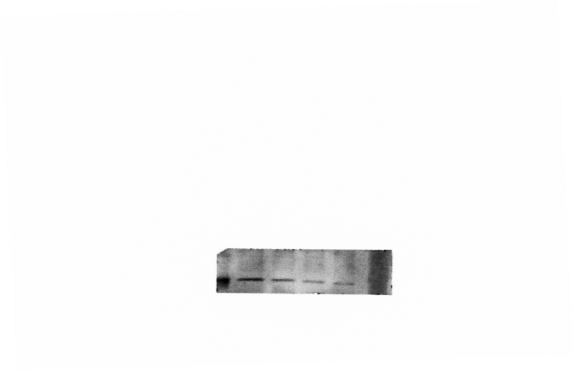

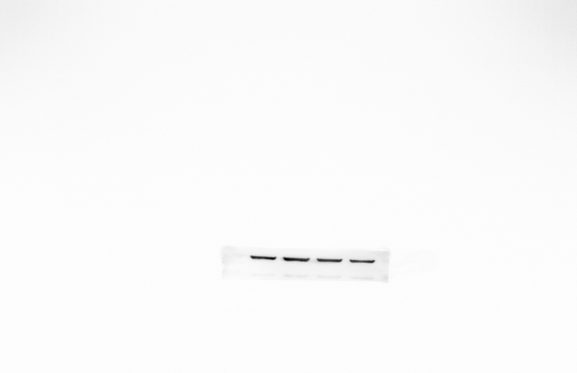
**

**Figure 2C-LN18-Survivin Figure 2C-LN18-β-actin**

**Western blot original images Figure 2D**

**
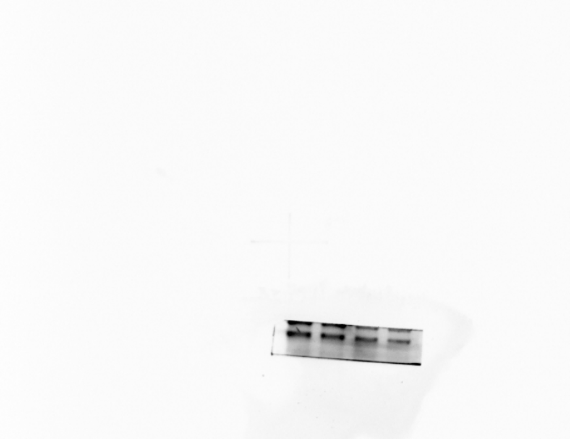

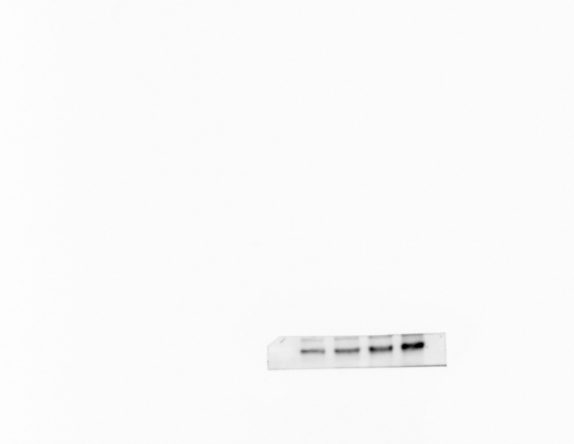
**

**Figure 2D-T98G-Caspase-3 Figure 2D-T98G-Cleaved-caspase-3**

**
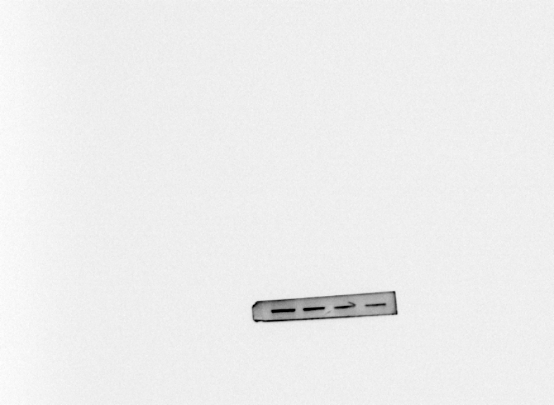

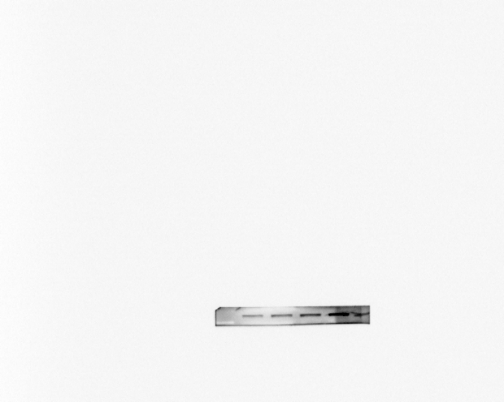
**

**Figure 2D-T98G-PARP-1 Figure 2D-T98G-Cleaved-PARP-1**

**
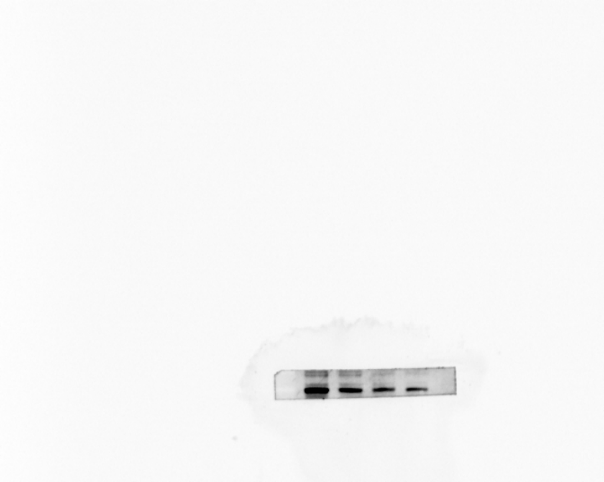

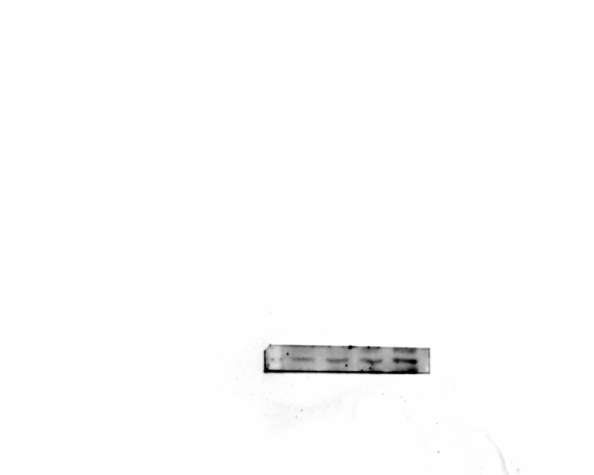
**

**Figure 2D-T98G-Caspase-9 Figure 2D-T98G-Cleaved-caspase-9**

**
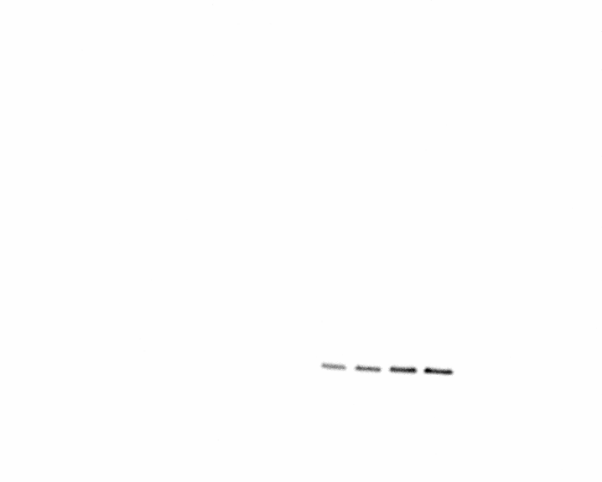

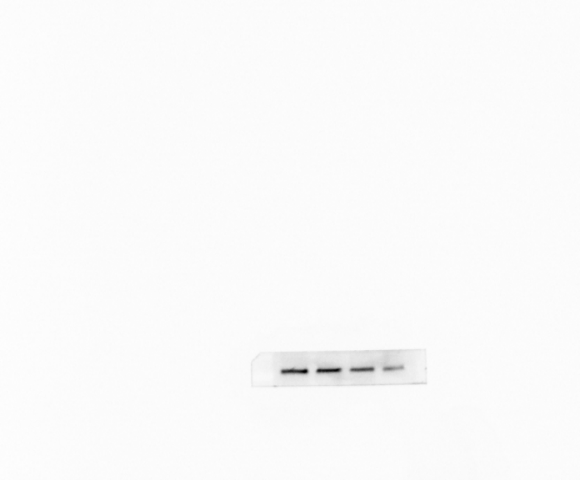
**

**Figure 2D-T98G-Bax Figure 2D-T98G-Bcl-2**

**
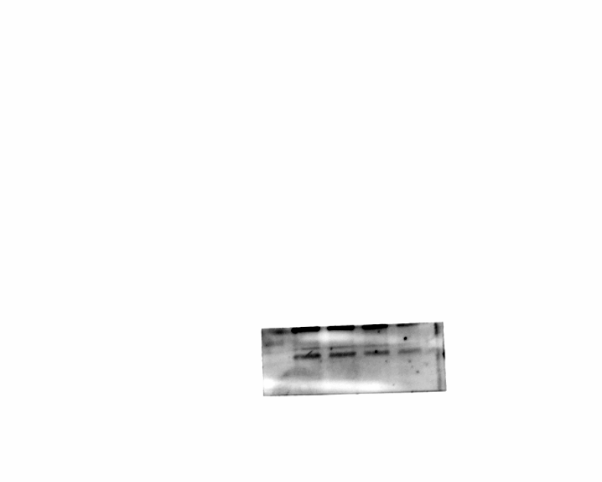

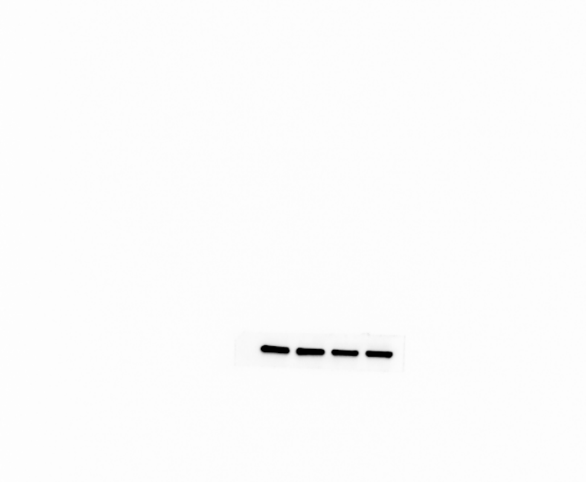
**

**Figure 2D-T98G-Survivin Figure 2D-T98G-β-actin**

**
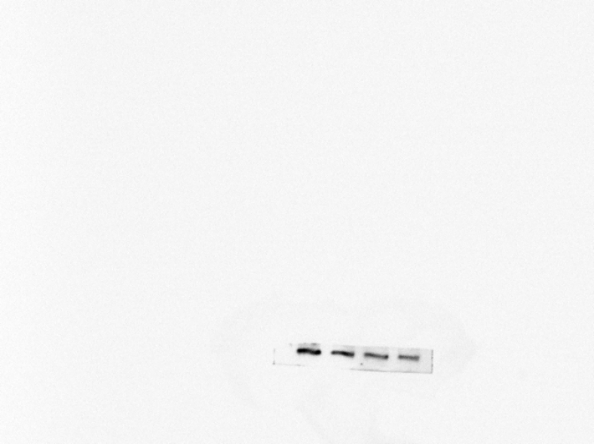

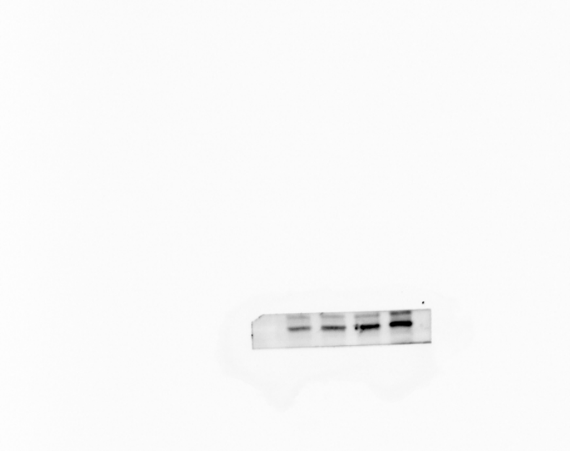
**

**Figure 2D-LN18-Caspase-3 Figure 2D-LN18-Cleaved-caspase-3**

**
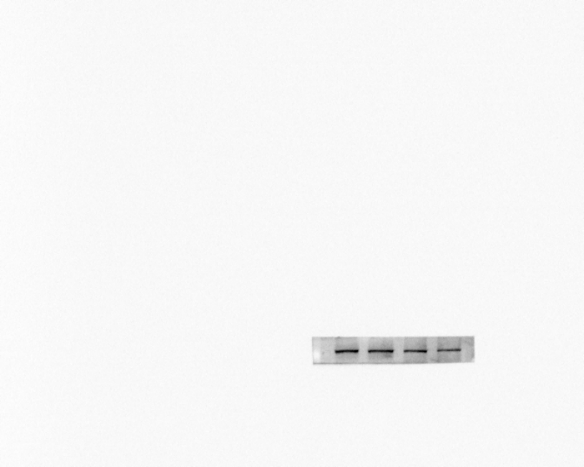

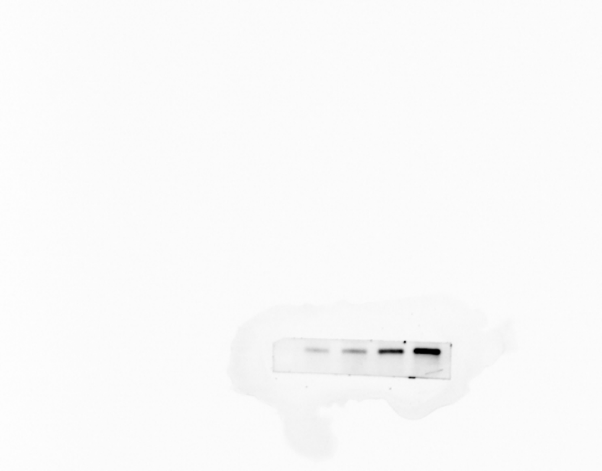
**

**Figure 2D-LN18-PARP-1 Figure 2D-LN18-Cleaved- PARP-1**

**
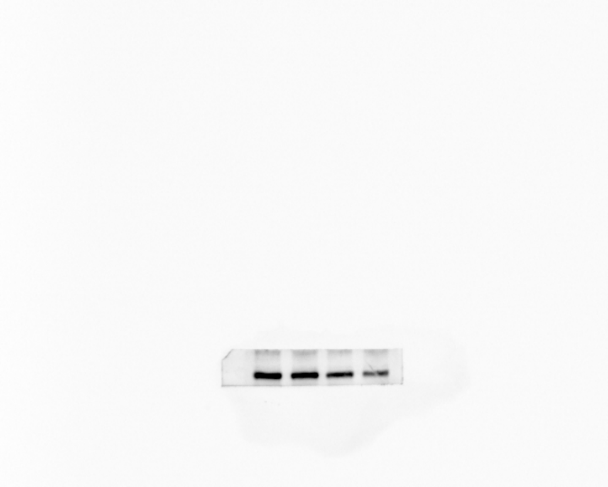

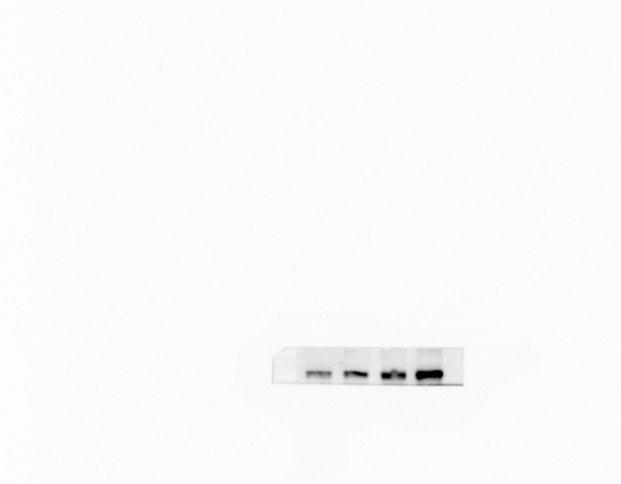
**

**Figure 2D-LN18-Caspase-9 Figure 2D-LN18-Cleaved-caspase-9**

**
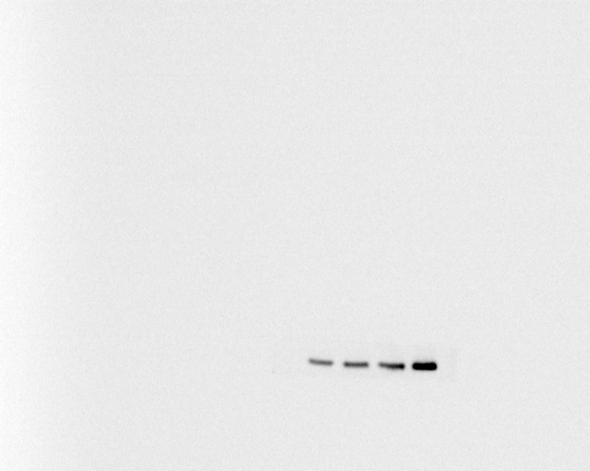

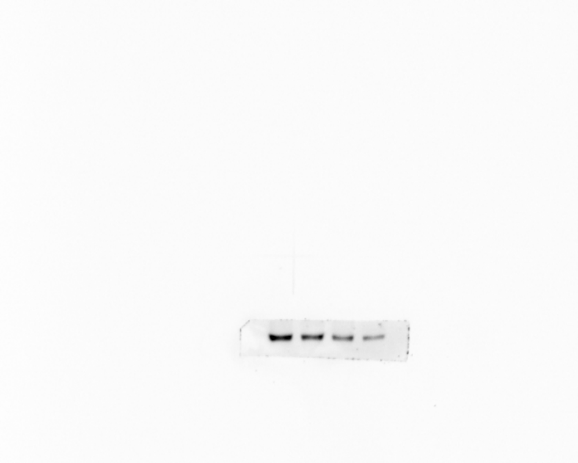
**

**Figure 2D-LN18-Bax Figure 2D-LN18-Bcl-2**

**
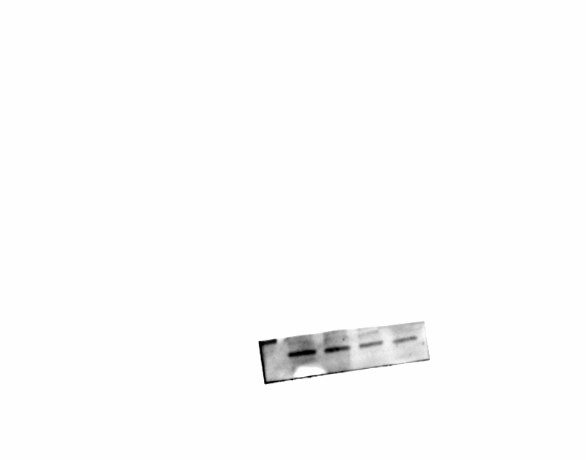

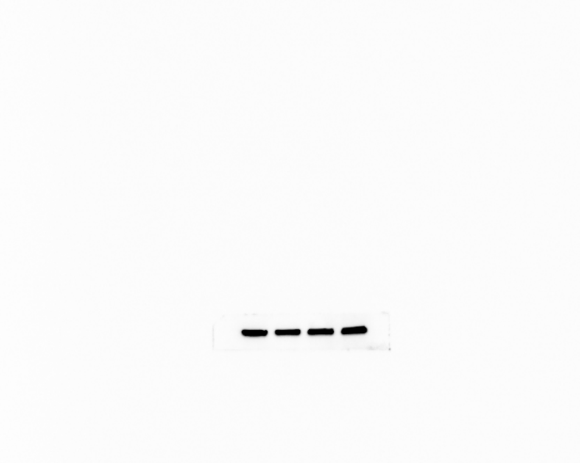
**

**Figure 2D-LN18-Survivin Figure 2D-LN18-β-actin**

**Western blot original images Figure 2F**

**
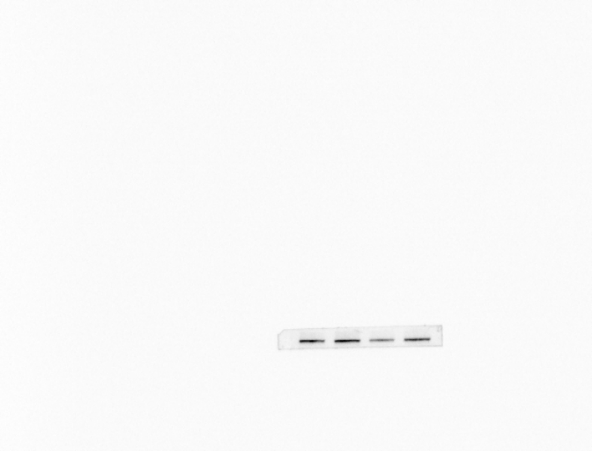

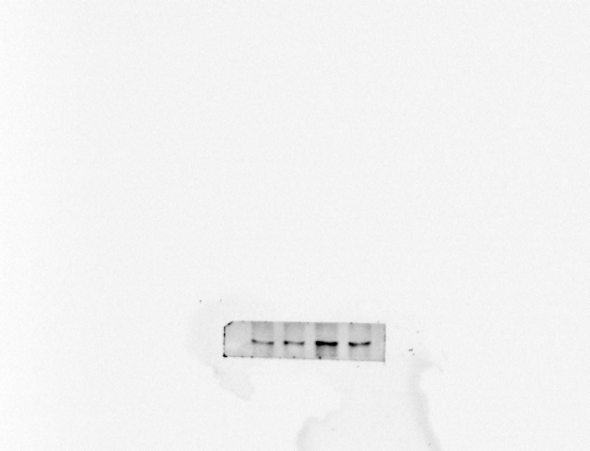
**

**Figure 2F-T98G-PARP-1 Figure 2F-T98G-Cleaved-PARP-1**

**
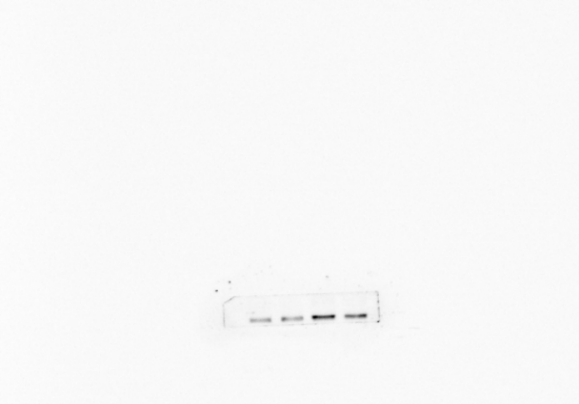

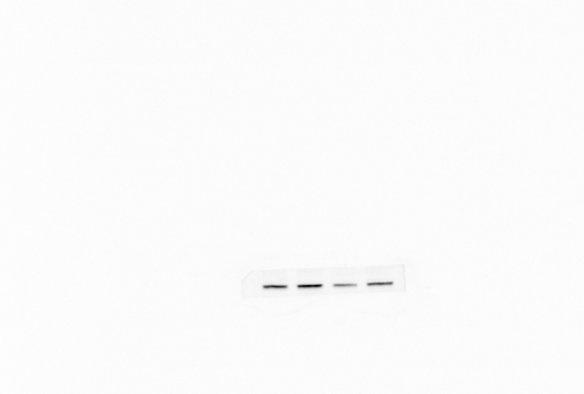
**

**Figure 2F-T98G-Cleaved-Caspase-9 Figure 2F-T98G -Caspase-9**

**
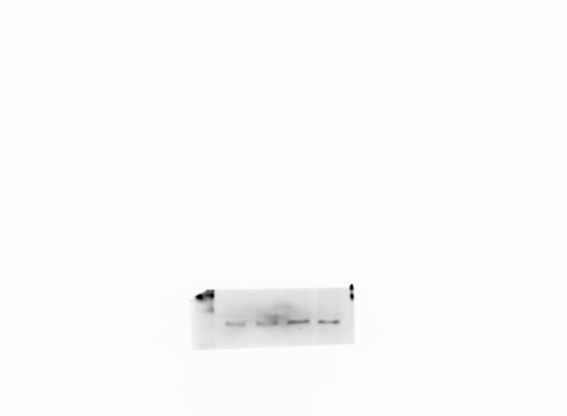

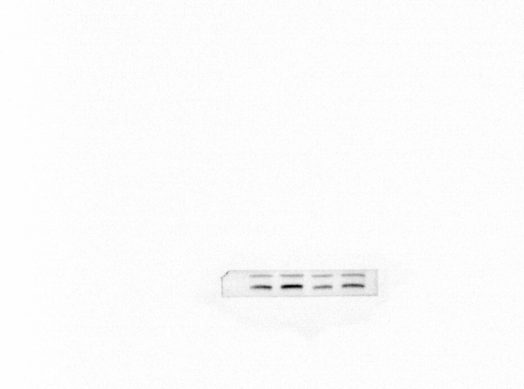
**

**Figure 2F-T98G- Cleaved Caspase-3 Figure 2F-T98G-Caspase-3**

**
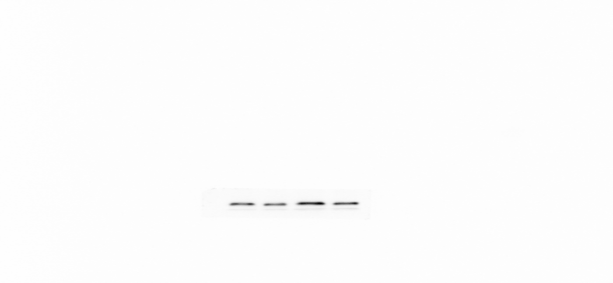

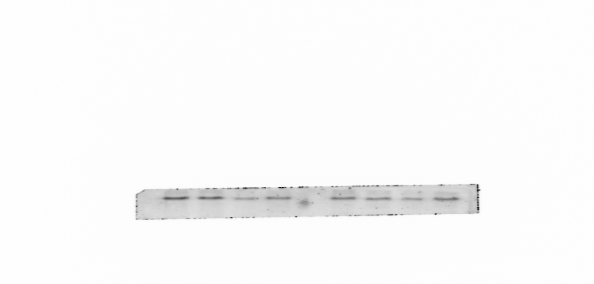
**

**Figure 2F-T98G-Bax Figure 2F-T98G- Bcl-2**

**
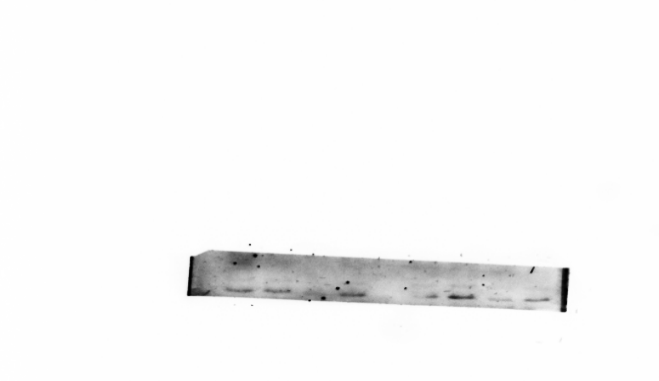

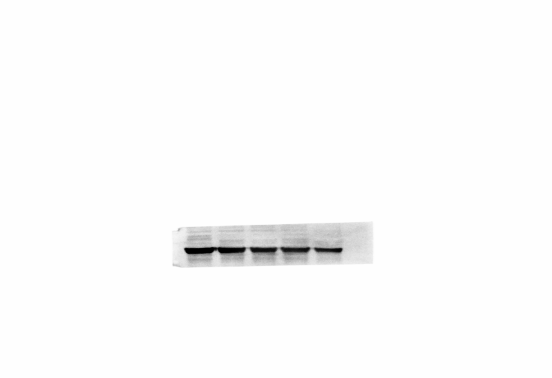
**

**Figure 2F-T98G-Survivin Figure 2F-T98G- β-actin**

**
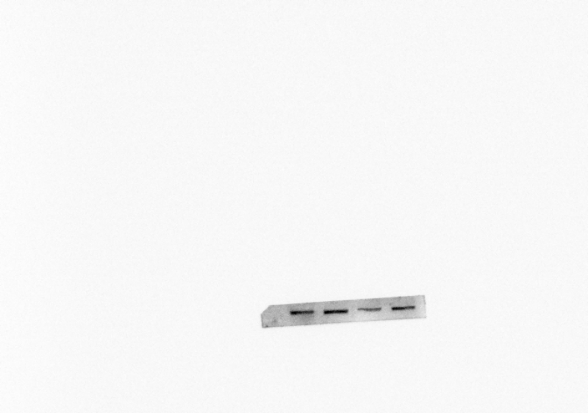

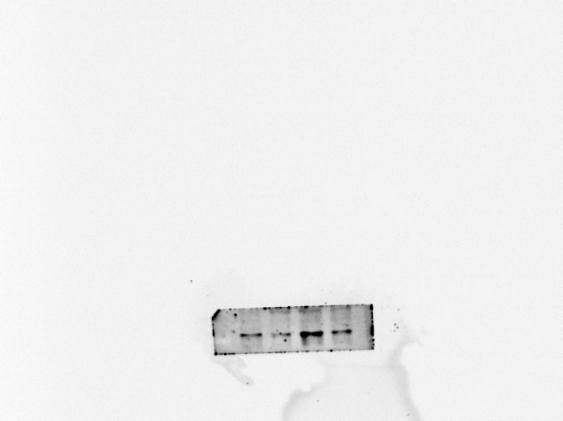
**

**Figure 2F-LN18-PARP-1 Figure 2F-LN18-Cleaved-PARP-1**

**
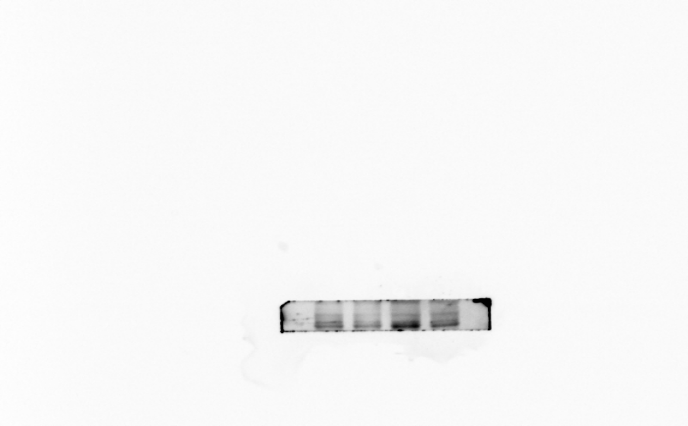

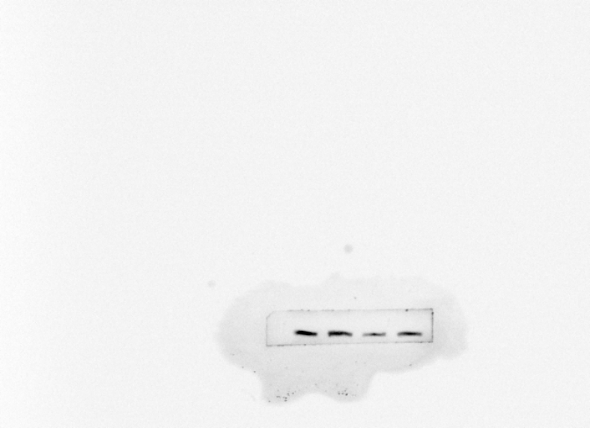
**

**Figure 2F-LN18-Cleaved-Caspase-9 Figure 2F-LN18-Caspase-9**

**
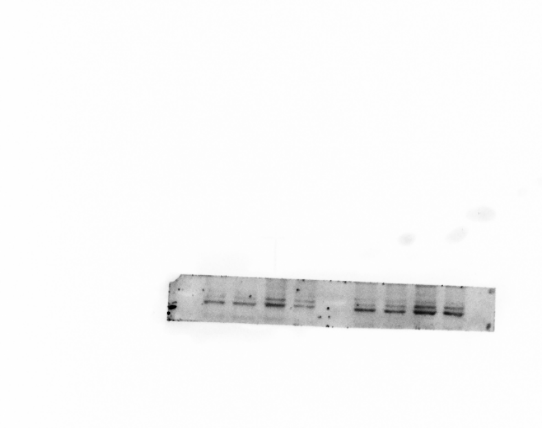

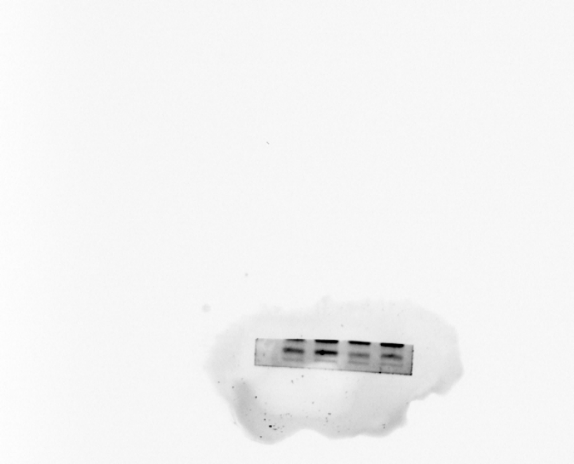
**

**Figure 2F-LN18- Cleaved Caspase-3 Figure 2F-LN18- Caspase-3**

**
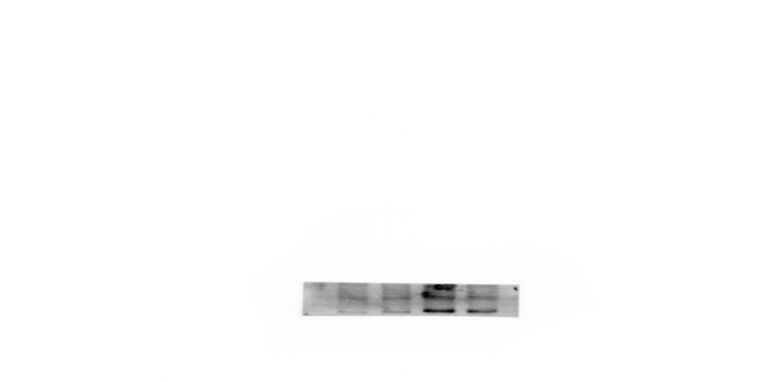

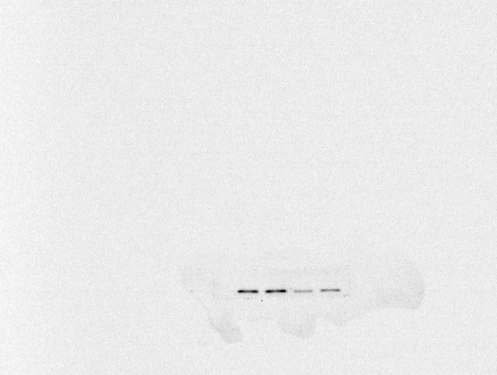
**

**Figure 2F-LN18-Bax Figure 2F-LN18- Bcl-2**

**
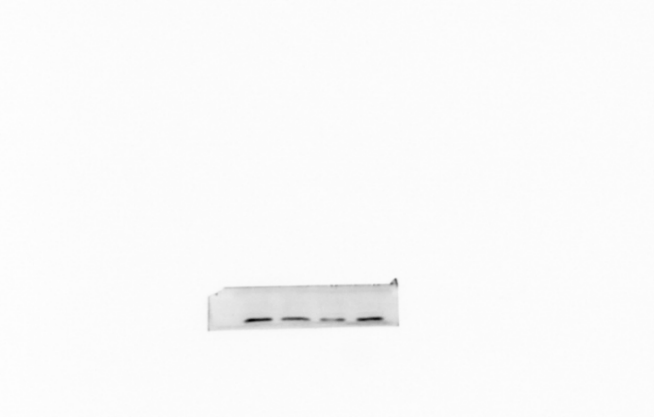

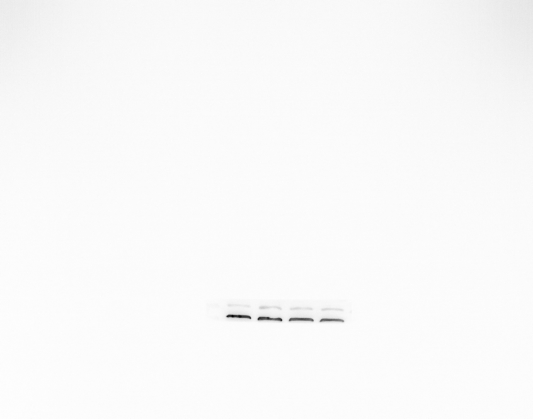
**

**Figure 2F-LN18-Survivin Figure 2F-LN18-β-actin**

**Western blot original images Figure 3E**

**
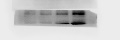

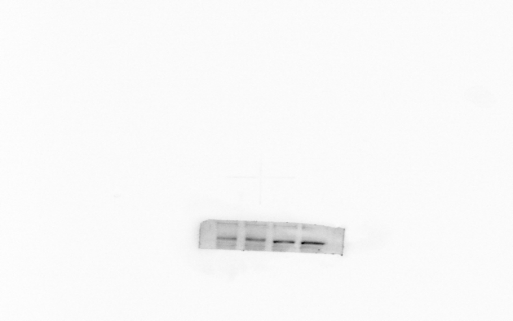
**

**Figure 3E-T98G- p-Histone H2A.X Figure 3E-T98G- Chk2**

**
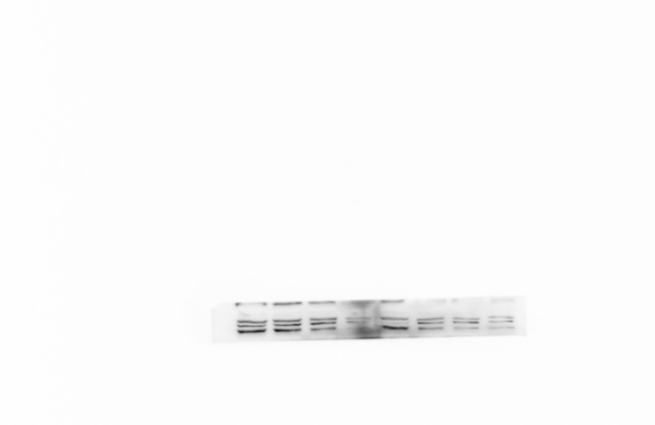

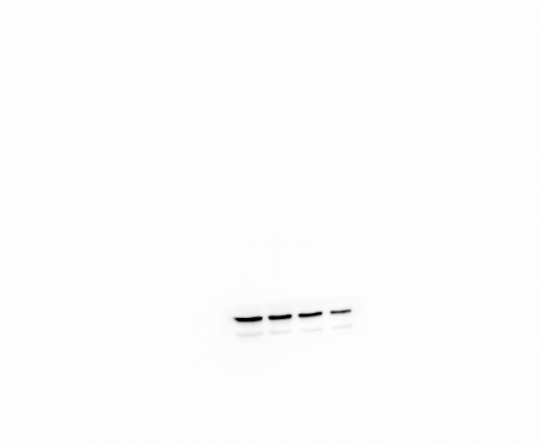
**

**Figure 3E-T98G- cdc25A Figure 3E-T98G- CyclinA2**

**
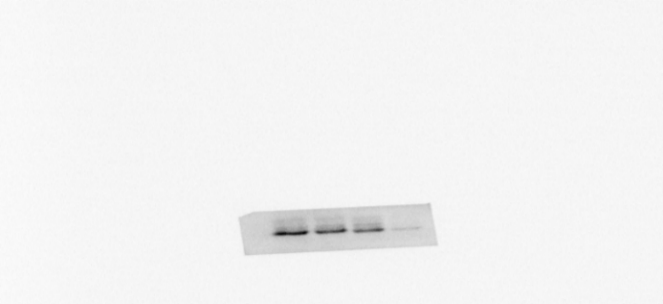

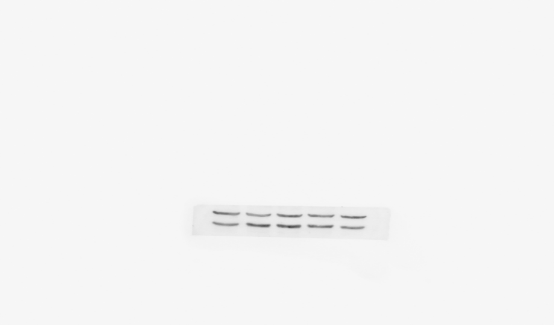
**

**Figure 3E-T98G- Cdk2 Figure 3E-T98G-β-actin**

**
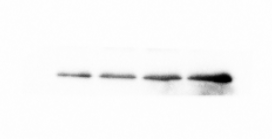

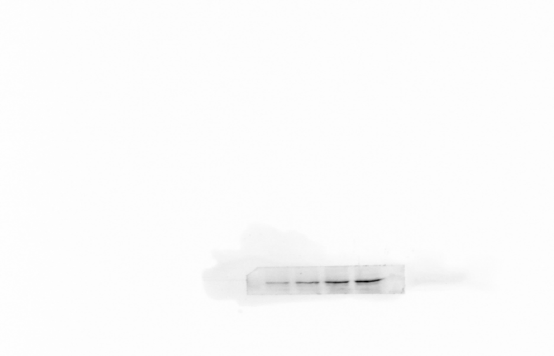
**

**Figure 3E-LN18- p-Histone H2A.X Figure 3E-LN18- Chk2**

**
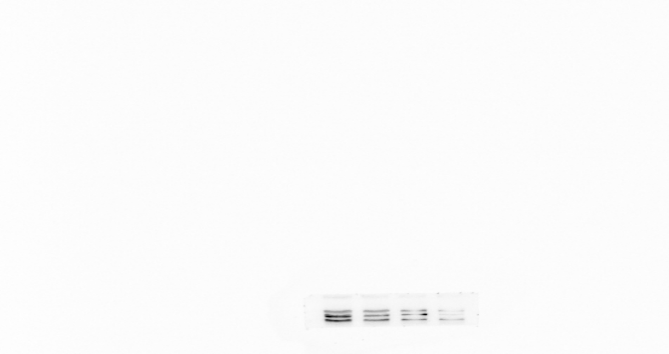

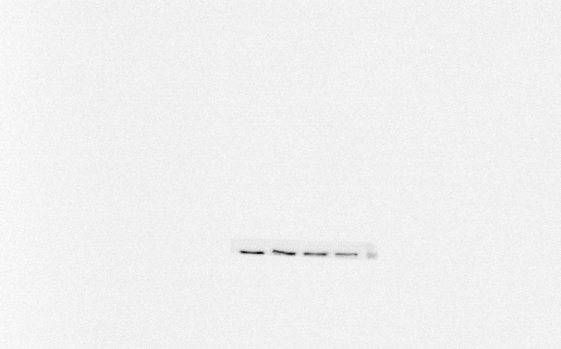
**

**Figure 3E-LN18- Cdc25A Figure 3E-LN18- CyclinA2**

**
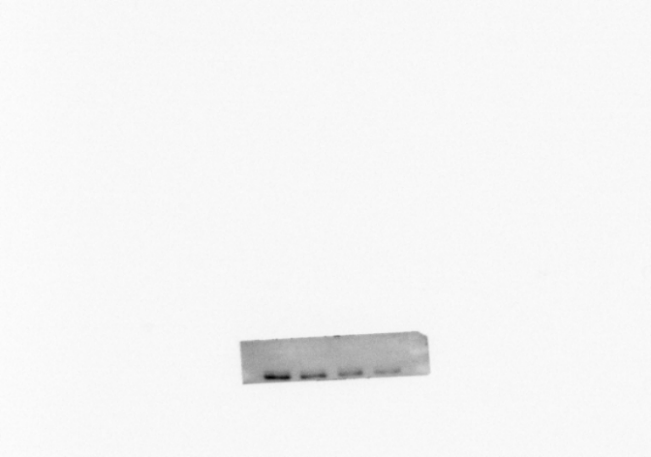

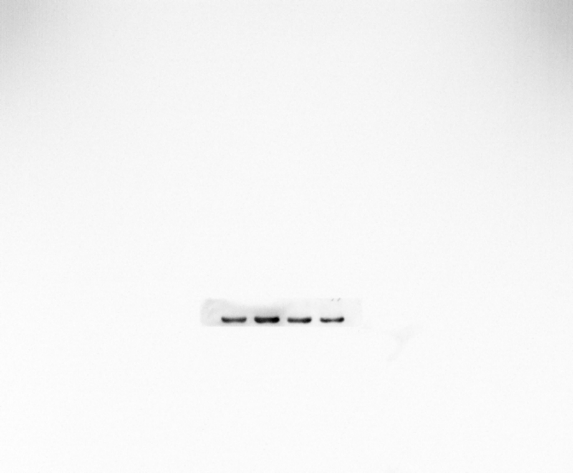
**

**Figure 3E-LN18-Cdk2 Figure 3E-LN18-β-actin**

**Western blot original images Figure 4E**

**
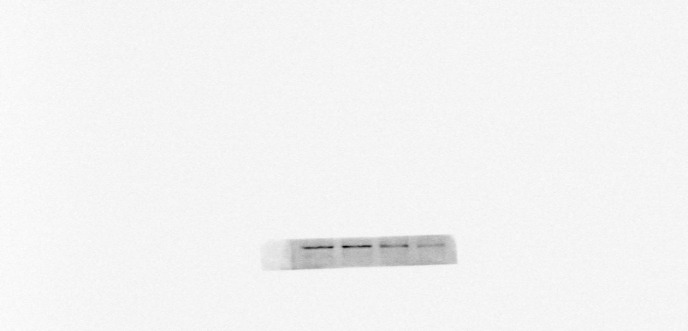

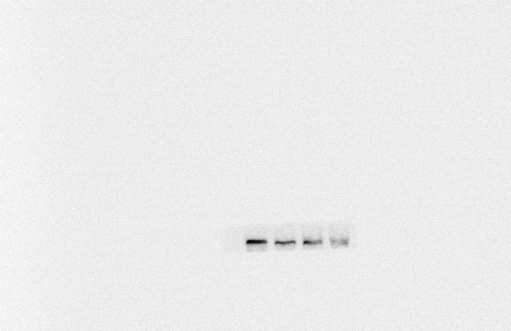
**

**Figure 4E-T98G-MMP9 Figure 4E-T98G- MMP2**

**
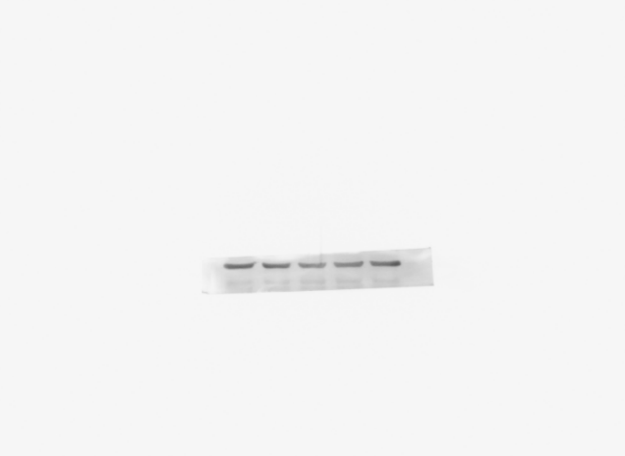

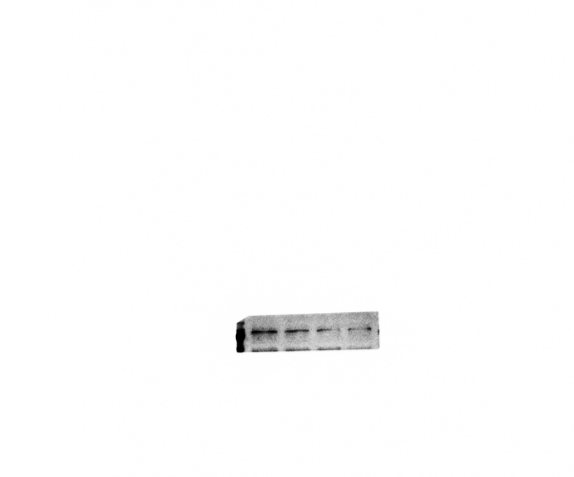
**

**Figure 4E-T98G-β-actin Figure 4E-LN18- MMP9**

**
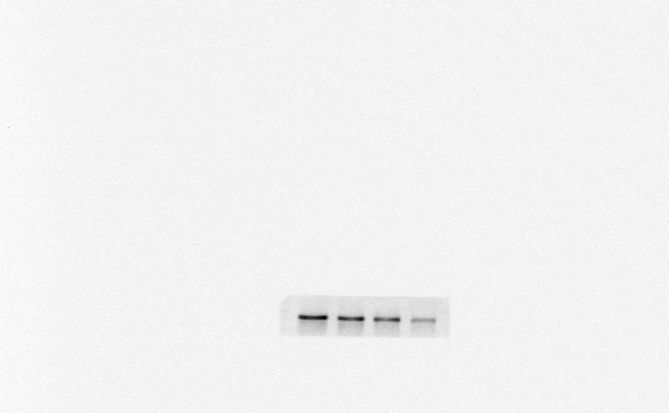

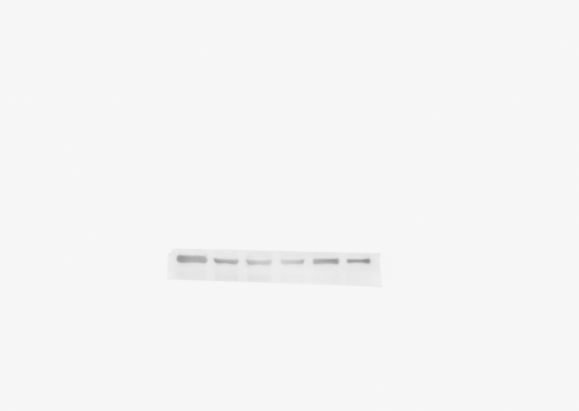
**

**Figure 4E-LN18- MMP2 Figure 4E-LN18-β-actin**

**Western blot original images Figure 4F**

**
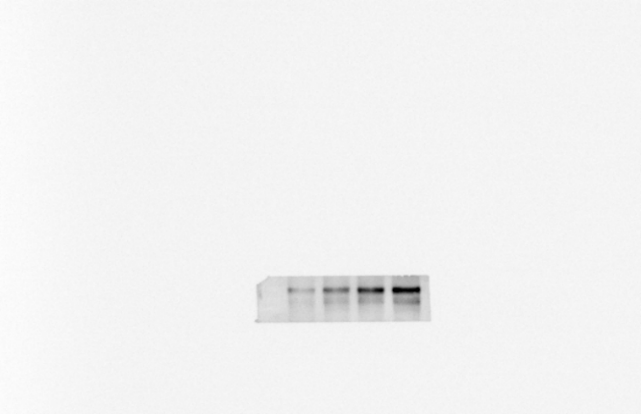

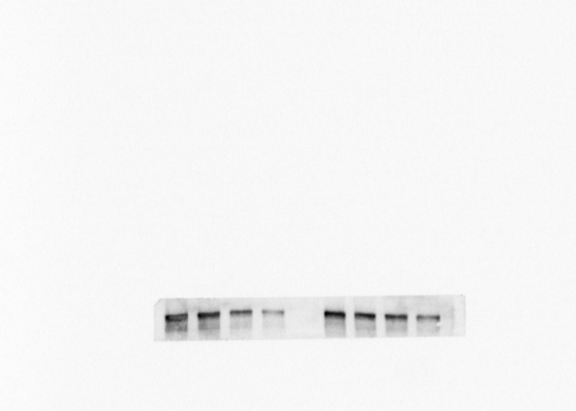
**

**Figure 4F-T98G-E-cadherin Figure 4F-T98G-N-cadherin**

**
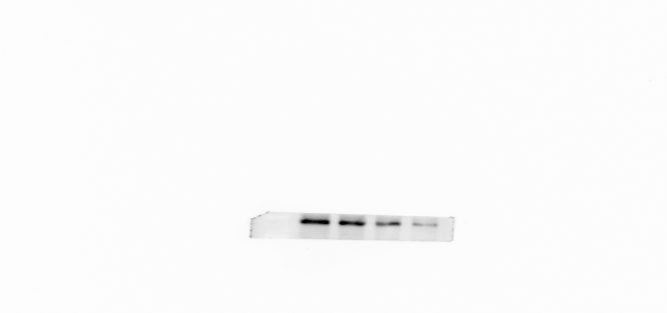

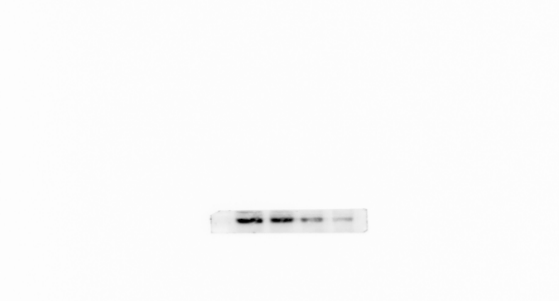
**

**Figure 4F-T98G-Slug Figure 4F-T98G-Snail**

**
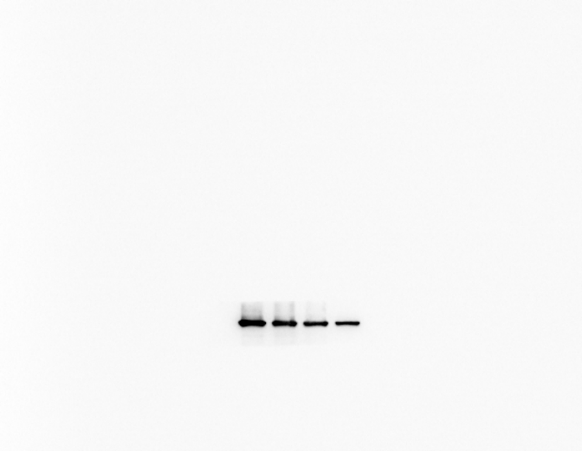

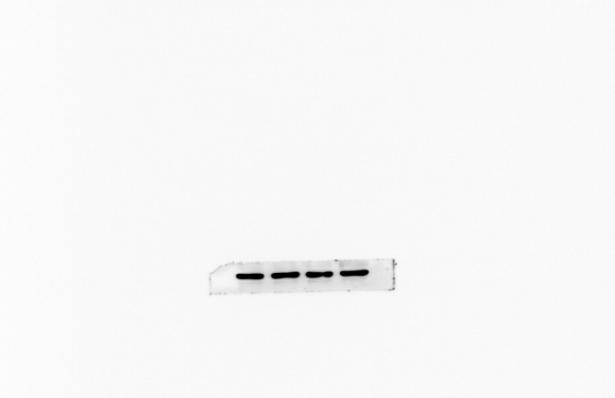
**

**Figure 4F-T98G-Vimentin Figure 4F-T98G-****β-actin**

**
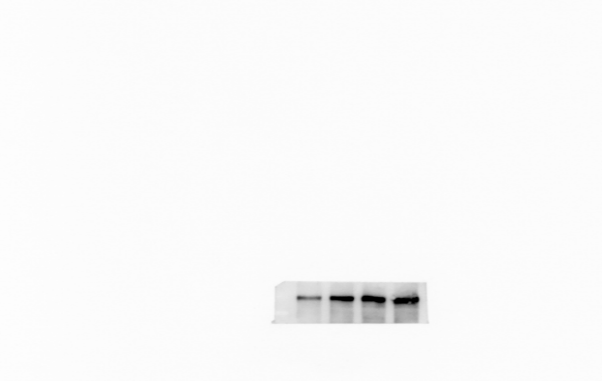

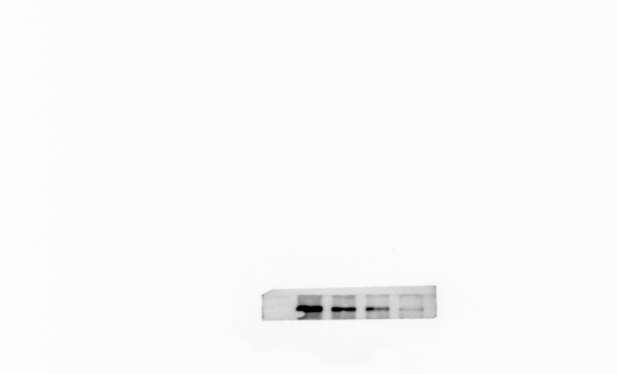
**

**Figure 4F-LN18-E-cadherin Figure 4F-LN18-N-cadherin**

**
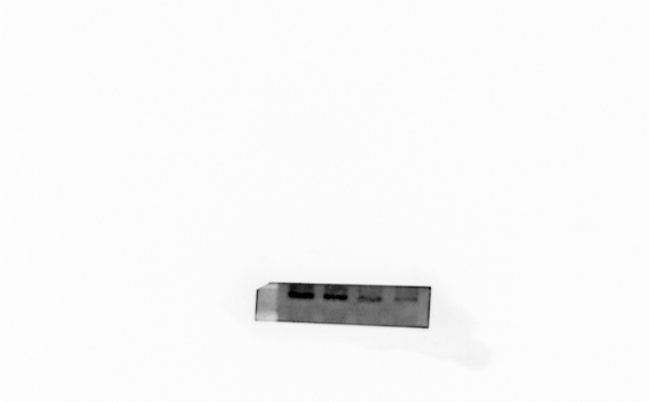

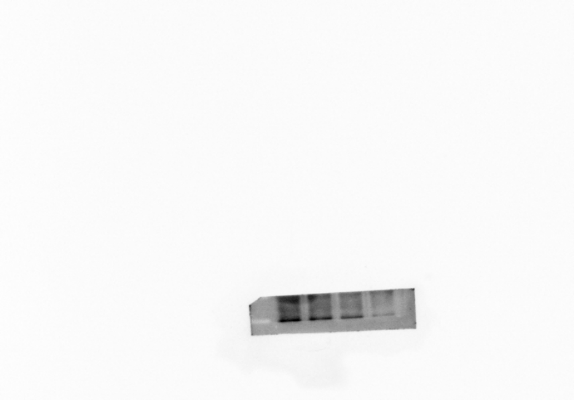
**

**Figure 4F-LN18-Slug Figure 4F-LN18-Snail**

**
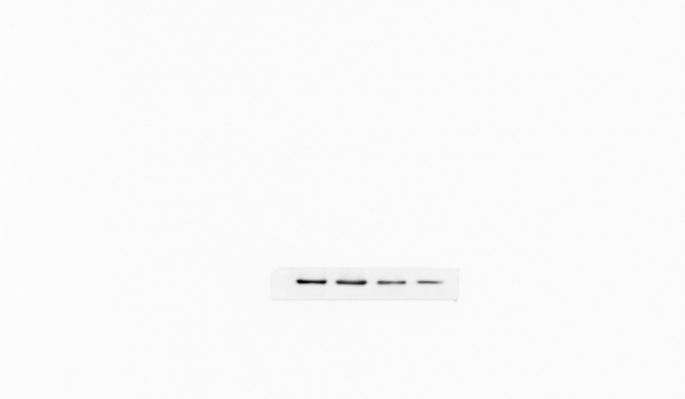

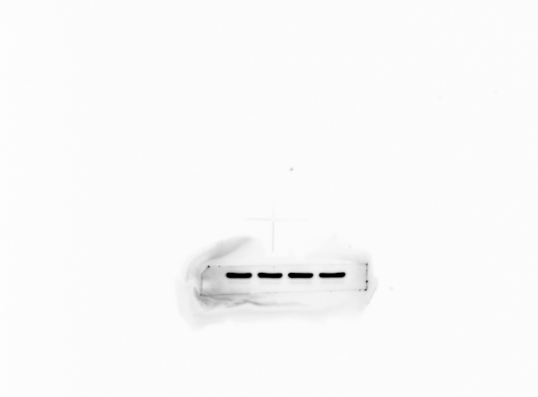
**

**Figure 4F-LN18-Vimentin Figure 4F-LN18-β-actin**

**Western blot original images Figure 6D**

**
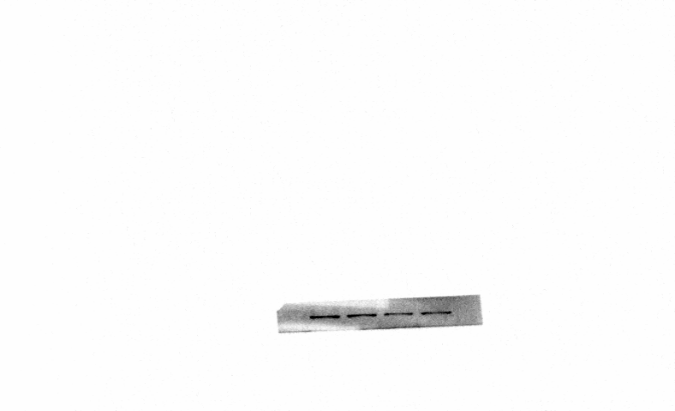

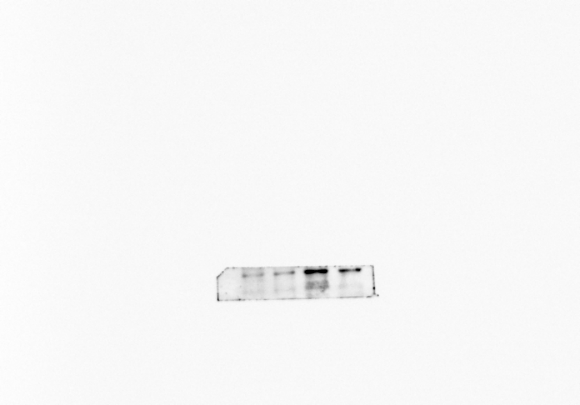
**

**Figure 6D-T98G-PARP-1 Figure 6D-T98G-Cleaved-PARP-1**

**
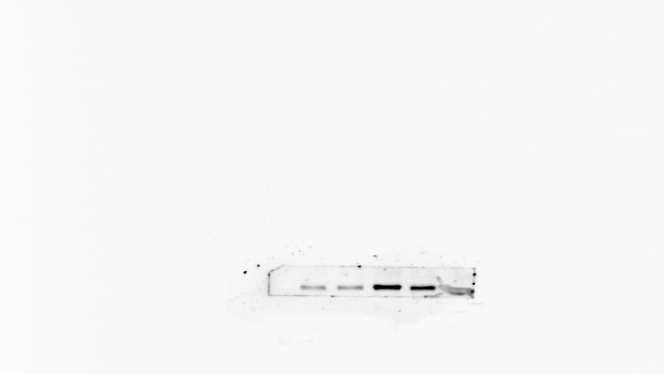

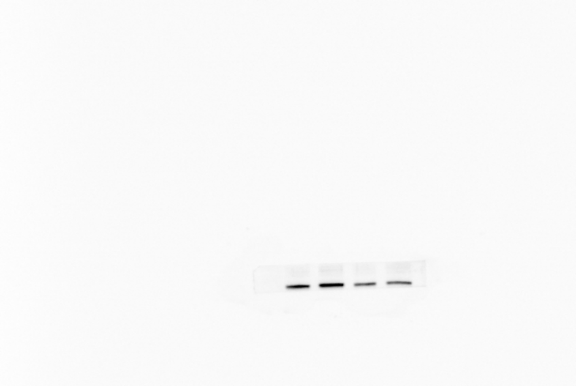
**

**Figure 6D-T98G-Cleaved-Caspase-9 Figure 6D-T98G-Caspase-9**

**
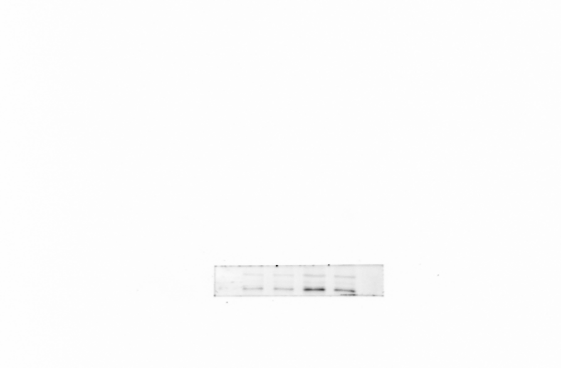

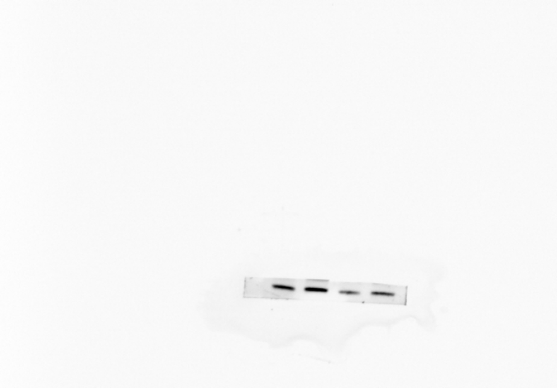
**

**Figure 6D-T98G Cleaved Capase-3 Figure 6D-T98G Capase-3**

**
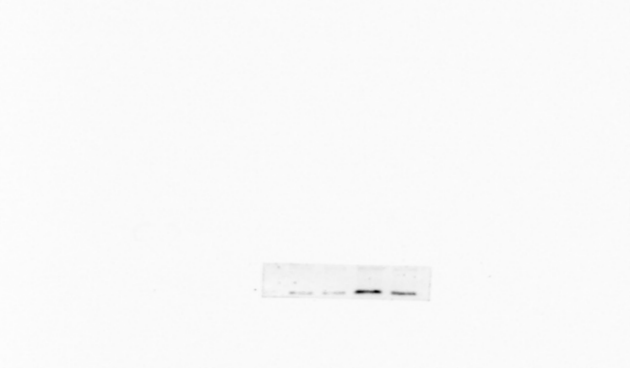

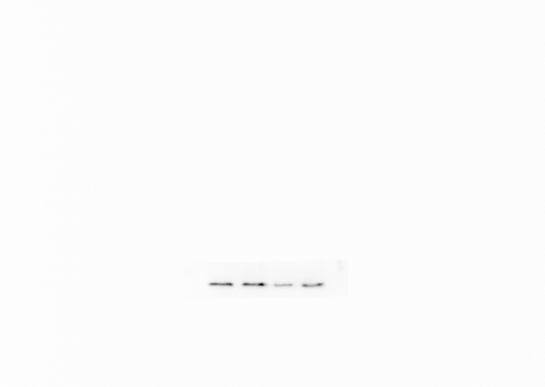
**

**Figure 6D-T98G-Bax Figure 6D-T98G-Bcl-2**

**
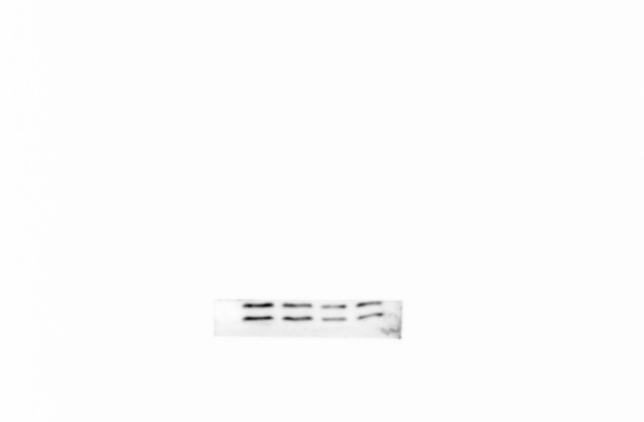

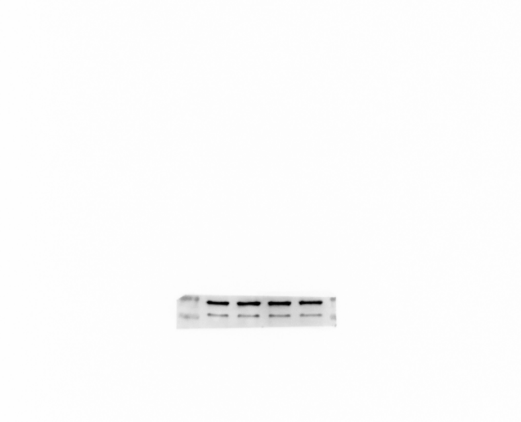
**

**Figure 6D-T98G-Survivin Figure 6D-T98G-β-actin**

**Figure 6D-LN18-PARP-1 Figure 6D-LN18-Cleaved-PARP-1**

**Figure 6D-LN18-Cleaved-Caspase-9 Figure 6D-LN18- Caspase-9**

**Figure 6D-LN18- Cleaved Caspase-3 Figure 6D-LN18-Caspase-3**

**Figure 6D-LN18-Bax Figure 6D-LN18- Bcl-2**

**Figure 6D-LN18-Survivin Figure 6D-LN18-β-actin**

**Western blot original images Figure 6F**

**Figure 6F-T98G- p-ERK Figure 6F-T98G-ERK**

**Figure 6F-T98G- p-JNK Figure 6F-T98G-JNK**

**Figure 6F-T98G- p-c-Jun Figure 6F-T98G- c-Jun**

**Figure 6F-T98G- p-p38 Figure 6F-T98G- p38**

**Figure 6F-T98G- β-actin Figure 6F-LN18- p-ERK**

**Figure 6F-LN18-ERK Figure 6F-LN18- p-JNK**

**Figure 6F-LN18-JNK Figure 6F-LN18- p-c-Jun**

**Figure 6F-LN18- c-Jun Figure 6F-LN18- p-p38**

**Figure 6F-LN18- p38 Figure 6F-LN18- β-actin**

**Western blot original images Figure 6G**

**Figure 6G-T98G-Cleaved Caspase-3 Figure 6G-T98G-Caspase-3**

**Figure 6G-T98G-Bax Figure 6G-T98G- Bcl-2**

**Figure 6G-T98G-Survivin Figure 6G-T98G-Caspase-9**

**Figure 6G-T98G-Cleaved-Caspase-9 Figure 6G-T98G-PARP-1**

**Figure 6G-T98G-Cleaved-PARP-1 Figure 6G-T98G-ERK**

**Figure 6G-T98G-p-ERK Figure 6G-T98G-JNK**

**Figure 6G-T98G-p-JNK** **Figure 6G-T98G- β-actin**

**Figure 6G-LN18- Cleaved Caspase-3 Figure 6G-LN18-Caspase-3**

**Figure 6G-LN18-Bax Figure 6G-LN18- Bcl-2**

**Figure 6G-LN18-Survivin Figure 6G-LN18-Caspase-9**

**Figure 6G-LN18-Cleaved-Caspase-9 Figure 6G-LN18-PARP-1**

**Figure 6G-LN18-Cleaved-PARP-1 Figure 6G-LN18-ERK**

**Figure 6G-LN18-p-ERK Figure 6G-LN18-JNK**

**Figure 6G-LN18-p-JNK Figure 6G-LN18- β-actin**
